# αvβ3 Integrin induces partial EMT independent of TGF-β signaling

**DOI:** 10.1038/s42003-021-02003-6

**Published:** 2021-04-21

**Authors:** Yoshinobu Kariya, Midori Oyama, Takato Suzuki, Yukiko Kariya

**Affiliations:** 1 Department of Biochemistry, Fukushima Medical University School of Medicine, Fukushima City, 960-1295 Japan; 2 Laboratory Animal Research Center, Fukushima Medical University School of Medicine, Fukushima City, 960-1295 Japan; 3 Department of Clinical Laboratory Medicine, Fukushima Medical University Hospital, Fukushima City, 960-1295 Japan

**Keywords:** Metastasis, Non-small-cell lung cancer, Cancer, Integrins

## Abstract

Epithelial–mesenchymal transition (EMT) plays a pivotal role for tumor progression. Recent studies have revealed the existence of distinct intermediate states in EMT (partial EMT); however, the mechanisms underlying partial EMT are not fully understood. Here, we demonstrate that αvβ3 integrin induces partial EMT, which is characterized by acquiring mesenchymal phenotypes while retaining epithelial markers. We found αvβ3 integrin to be associated with poor survival in patients with lung adenocarcinoma. Moreover, αvβ3 integrin-induced partial EMT promoted migration, invasion, tumorigenesis, stemness, and metastasis of lung cancer cells in a TGF-β-independent fashion. Additionally, TGF-β1 promoted EMT progression synergistically with αvβ3 integrin, while a TGF-β signaling inhibitor showed no effect on αvβ3 integrin-induced partial EMT. Meanwhile, the microRNA-200 family abolished the αvβ3 integrin-induced partial EMT by suppressing αvβ3 integrin cell surface expression. These findings indicate that αvβ3 integrin is a key inducer of partial EMT, and highlight a new mechanism for cancer progression.

## Abstract (web summary)

Kariya, Oyama et al. propose that αvβ3 can drive a partial EMT phenotype characterized by maintained E-cadherin expression and upregulation of mesenchymal markers. This hybrid EMT state is independent of TGF-β1 signaling and characterised by increased migration, invasion, tumor cell proliferation, stemness and metastatic capacity.

## Introduction

Lung cancer is the leading cause of cancer incidence and mortality worldwide^1,2^. Non-small cell lung cancer (NSCLC) accounts for approximately 85% of all lung cancers and has a poor overall 5-year survival rate (~15%)^3^. This poor prognosis is largely due to locally advanced or distant metastatic tumors at the time of diagnosis^4^. Although efficient targeted therapies and immunotherapies have been developed for NSCLC treatment over the past two decades, only 15–20% of NSCLC patients benefit from these therapies, and their efficacy is constrained by the emergence of drug-resistant cancers^5,6^. Therefore, it is important to further identify novel driver genes associated with tumor progression in lung cancer, as well as to understand the underlying molecular mechanisms, in order to overcome this disease and develop novel cancer therapies.

Epithelial-to-mesenchymal transition (EMT) is a cellular process in which cells lose their polarity and cell–cell contact, and acquire motile mesenchymal phenotype via cytoskeletal reorganization. In cancer, EMT is associated with tumorigenesis, invasion, and metastasis^7^. The regulation of EMT is mediated by several EMT-related transcriptional factors and microRNAs, including the miR-200 family (miR-200a, miR-200b, miR-200c, miR-141, and miR-429)^8^. Recent studies have found that EMT is not a binary process, but includes multiple intermediate states between epithelial and mesenchymal phenotypes, known as a partial EMT. Indeed, multiple tumor subpopulations have distinct partial EMT states^9,10^. Recently, the partial EMT has been recognized to possess higher motility, drug resistance, and tumor-initiating potential than complete EMT^10,11^. However, the molecular mechanisms that lead to a partial EMT state remain unclear, as do the implications of the hybrid phenotype for cancer progression.

Integrins are heterodimeric transmembrane glycoproteins consisting of α and β integrin subunits. β3 integrin forms a heterodimer with αv integrin and is expressed as αvβ3 integrin in tumor tissues. αvβ3 integrin is a cell-surface receptor for the Arg-Gly-Asp (RGD) sequence-containing extracellular matrix (ECM), such as fibronectin, vitronectin, and osteopontin. The expression levels of αvβ3 integrin are low in healthy epithelial tissues but high in tumor tissues^12^. High expression of αvβ3 integrin is correlated with tumor growth, cancer invasion, metastasis, and stemness^13–15^. Therefore, αvβ3 integrin is thought to play a central role in cancer progression^12,16^.

αvβ3 integrin has been shown to be involved in transforming growth factor-β (TGF-β)-induced EMT^17,18^. TGF-β is well known as a potent inducer of EMT in advanced cancer, and thereby promotes tumorigenesis and metastasis^19^. Meanwhile, EMT is induced by not only TGF-β but also various extracellular stimuli, such as ECM components and hypoxia, as well as cytokines. In several types of human cancer cells, including NSCLC cells, reduced expression of type II TGF-β receptor, which is required for TGF-β signaling, has been reported. This reduced expression has been associated with more aggressive tumor behavior^20–22^. These observations raise the possibility that other extracellular signals may induce distinct EMT states of tumor cells from TGF-β signaling. In the present study, our data indicate that αvβ3 integrin induces a partial EMT distinct from EMT induced by TGF-β signaling in lung cancer cells, and that this partial EMT is implicated in tumor progression.

## Results

### β3 Integrin is upregulated and associated with poor prognosis in lung adenocarcinoma

**Fig. 1:**
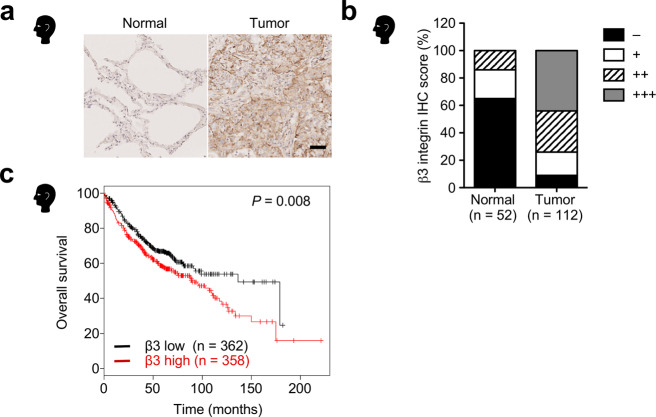
Upregulation of β3 integrin in lung adenocarcinoma is associated with poor survival. **a** Representative immunohistochemistry images of β3 integrin expression in human normal lung and lung adenocarcinoma tumor tissues. Scale bar, 50 µm. **b** β3 integrin expression in human normal lung tissues (*n* = 52) versus lung adenocarcinoma tumor tissues (*n* = 112). The staining intensity was graded as follows: − (negative), + (weak), ++ (moderate), and +++ (strong). The source data are provided in Supplementary Data 1. **c** Kaplan-Meier analysis of the correlation between β3 integrin expression and overall survival for lung adenocarcinoma patients (*n* = 720) using the database Kaplan-Meier Plotter. *P* values were calculated using a log-rank test.

To explore the role of αvβ3 integrin in lung cancer, we first performed immunohistochemical analyses of β3 integrin in 112 lung adenocarcinoma tumor tissue samples and 52 normal lung tissue samples. Compared with normal lung tissues, β3 integrin expression was upregulated in the lung adenocarcinoma tumor tissues (Fig. 1a, b). We next assessed whether β3 integrin expression was correlated with poor patient survival in lung adenocarcinoma. Kaplan-Meier analysis of a dataset comprising 720 patients with lung adenocarcinoma revealed that high expression of β3 integrin showed a significant reduction in patient survival (Fig. 1c). Collectively, these data suggest that upregulation of β3 integrin is associated with malignant progression in lung adenocarcinoma.

### αvβ3 Integrin is involved in partial EMT

**Fig. 2:**
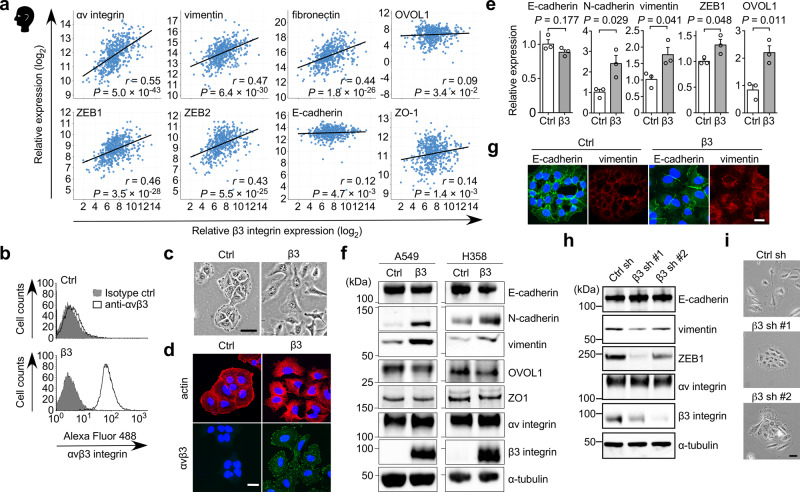
Identification of αvβ3 integrin as a partial EMT inducer. **a** Correlation analysis between β3 integrin and the indicated EMT-related gene expression levels in human lung adenocarcinoma tissues by RNA sequencing from TCGA dataset. Spearman’s rank correlation and *P* value are shown (*n* = 517 patients). **b** FACS analysis of the cell surface expression of αvβ3 integrin in control and β3 integrin expressing A549 cells. **c** Cell morphology of control and β3-A549 cells. Scale bar, 50 µm. **d** F-actin (red) and αvβ3 integrin (green) staining of control and β3-A549 cells. Images are shown with nuclear stain (blue). Scale bar, 25 µm. **e** Relative mRNA expression levels (qRT-PCR) of EMT markers in control and β3-A549 cells. Two-tailed unpaired Student’s *t*-test, mean ± s.e.m., *n* = 3 independent experiments conducted in triplicate. The source data are provided in Supplementary Data 1. **f** Western blotting of EMT markers in control and β3-A549 cells or control and β3-H358 cells. **g** Immunofluorescence-based single cell analysis using anti-E-cadherin and vimentin antibodies in control and β3-A549 cells. **h** Western blotting of EMT markers in H1975 cells expressing a control shRNA or two β3 integrin-specific shRNAs. **i** Cell morphology of H1975 cells expressing a control shRNA or two β3 integrin-specific shRNAs. Scale bar, 50 µm. Unprocessed blot images in (**f** and **h**) are shown in Supplementary Figs. 3–5.

EMT is highly associated with tumor progression^7,23^. To explore a possible role for β3 integrin upregulation in the pathological progression of lung adenocarcinoma, we performed a correlation analysis between β3 integrin and EMT markers using The Cancer Genome Atlas (TCGA) database. We found that β3 integrin correlated positively with mesenchymal markers vimentin, fibronectin, ZEB1, and ZEB2, as well as αv integrin (Fig. 2a). In contrast, there was almost no correlation between β3 integrin and the epithelial markers E-cadherin, OVOL1 and ZO-1 (Fig. 2a). To further investigate the effect of β3 integrin expression on EMT induction, we overexpressed β3 integrin in A549 (β3-A549), H358 (β3-H358), and H460 (β3-H460) cells, all of which are human lung cancer cell lines with αv but little β3 integrin expression^24,25^. Introduction of β3 integrin in the A549, H358, and H460 cells induced cell surface expression of αvβ3 integrin, elongation of cell bodies, and stress fiber rearrangement (Fig. 2b–d and Supplementary Fig. 1). In agreement with the correlation analysis between β3 integrin and EMT markers, β3 integrin-overexpressing lung cancer cells showed increased mesenchymal markers, including N-cadherin, vimentin, and ZEB1 (Fig. 2e, f); however, only small reductions were observed in E-cadherin, OVOL1, and ZO-1 (Fig. 2f). An immunofluorescence-based single cell analysis showed that β3-A549 cells were hybrid epithelial/mesenchymal cells, because the cells clearly expressed both E-cadherin and vimentin (Fig. 2g). In contrast, β3 integrin knockdown in H1975 cells, a human lung cancer cell line with endogenous αvβ3 integrin expression (Fig. 2h) and partial EMT phenotype^26^, led to the formation of colonies with epithelial morphology (Fig. 2i), decreased expression of vimentin and ZEB1, and slightly increased expression of E-cadherin (Fig. 2h). These data indicate that αvβ3 integrin expression induces partial EMT state, exhibiting both epithelial and mesenchymal phenotypes.

### αvβ3 Integrin-induced partial EMT accelerates migration, invasion, tumorigenesis, stemness, and metastasis

**Fig. 3:**
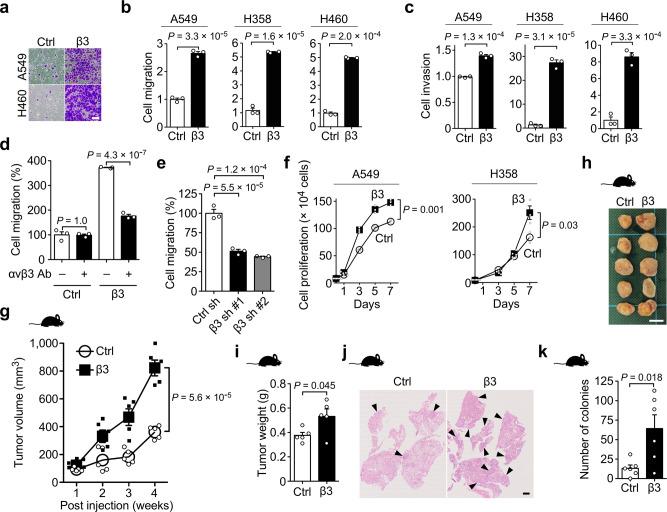
Enhancement of migration, invasion, tumorigenicity, and metastasis by αvβ3 integrin. **a** Representative images of migrated control and β3-A549 cells or control and β3-H460 cells in Boyden chamber assays. Scale bar, 100 µm. **b**, **c** Effect of αvβ3 integrin expression on the cell migration (**b**) and invasion (**c**) of A549, H358, and H460 cells. Two-tailed unpaired Student’s *t*-test, mean ± s.e.m. of three independent assays conducted in triplicate. **d**, **e** Effect of anti-αvβ3 integrin antibody (**d**) and β3 integrin-specific shRNA knockdown (**e**) on the cell migration of A549 (**d**) and H1975 (**e**) cells, respectively. One-way ANOVA and Bonferroni post-hoc test, mean ± s.e.m. of three independent assays conducted in triplicate. **f** Cell proliferation assays of control and β3-A549, or control and β3-H358 cells. Two-tailed unpaired Student’s *t*-test, mean ± s.e.m. of three independent assays conducted in quadruplicate. **g** Tumor growth of control and β3-A549 cells in nude mice. Two-tailed unpaired Student’s *t*-test, mean ± s.e.m., *n* = 5 mice per group. **h** Representative picture from the tumors of control and β3-A549 cells in nude mice at 4 weeks. Scale bar, 1 cm. **i** Primary tumor mass of control and β3-A549 cells in nude mice at 4 weeks. Two-tailed unpaired Student’s *t*-test, mean ± s.e.m., *n* = 5 mice per group. **j**, **k** Representative pictures from H&E-stained lungs (**j**), and quantification of lung colonies (**k**) at 7 weeks after tail vein injection of control and β3-A549 cells in nude mice. Arrowheads indicate tumor colonies. Scale bar, 1 mm. Two-tailed unpaired Student’s *t*-test, mean ± s.e.m., *n* = 6 mice per group. The source data are provided in Supplementary Data 1.

**Fig. 4:**
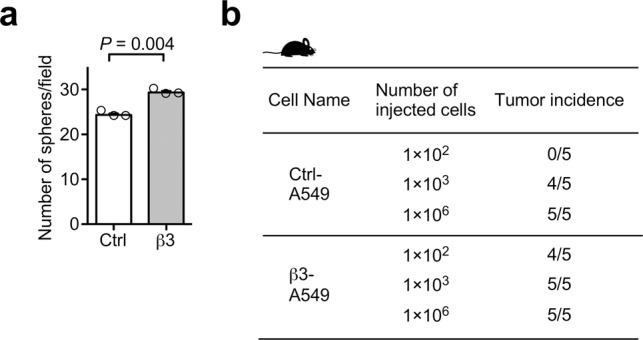
Enhancement of cancer stemness by αvβ3 integrin expression. **a** In vitro sphere assay of control and β3-A549 cells. Two-tailed unpaired Student’s *t*-test, mean ± s.e.m. of three independent assays conducted in triplicate. The source data are provided in Supplementary Data 1. **b** In vivo tumor initiation assay at 4 weeks after subcutaneous injection of the indicated number of control and β3-A549 cells in nude mice. *n* = 5 mice per group.

To investigate the functions of αvβ3 integrin-induced partial EMT in lung cancer progression, we first conducted transwell cell migration and Matrigel invasion assays to assess the functions of αvβ3 integrin in cell motility and invasiveness. The partial EMT markedly enhanced cell migration and invasion in A549, H358, and H460 cells (Fig. 3a–c). The increased cell motility was abrogated by anti-αvβ3 integrin antibody treatment (Fig. 3d). Consistent with these results, β3 integrin knockdown reduced the cell migration of H1975 cells that endogenously expressed αvβ3 integrin (Fig. 3e). Next, we examined the effect of αvβ3 integrin-induced partial EMT in lung cancer cells on tumor development (Figs. 3f–i and 4, and Supplementary Fig. 2). Expression of αvβ3 integrin in A549 cells significantly enhanced in vitro cell proliferation and sphere formation (Figs. 3f and  4a), as well as in vivo tumorigenesis, stemness, and cell proliferation (Figs. 3g–i and 4b, and Supplementary Fig. 2b) compared with the control cells. The immunohistochemical analysis of the tumors arising from β3-A549 cells using anti-E-cadherin and anti-vimentin antibodies revealed that β3-A549 cells maintained a partial EMT state in the tumors (Supplementary Fig. 2c). To determine whether αvβ3 integrin-induced partial EMT enhances in vivo metastatic ability, we injected control A549 and β3-A549 cells intravenously into the tail vein of nude mice, and evaluated their ability to cause metastasis. Exogenous expression of αvβ3 integrin markedly increased the lung metastatic colonization of the A549 cells (Fig. 3j, k). These results suggest that αvβ3 integrin-induced partial EMT promotes tumorigenesis and metastasis.

**Fig. 5:**
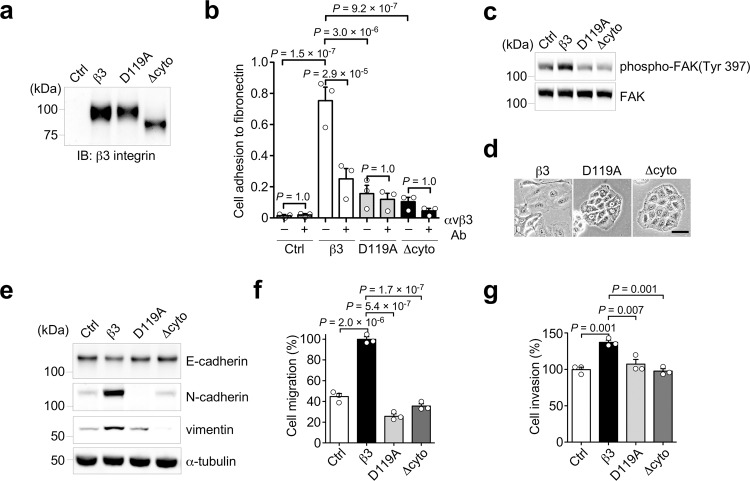
αvβ3 integrin signaling is required for its inducible partial EMT. **a** Western blotting of β3 integrin in control, β3, D119A β3, and ∆cyto β3 integrin-expressing A549 cells. **b** Cell adhesion of control, β3, D119A β3, and ∆cyto β3 integrin-expressing A549 cells to fibronectin, and the inhibitory effect of αvβ3 integrin antibody on the cell adhesion. **c** Phosphorylation of FAK in control, β3, D119A β3, and ∆cyto β3-expressing A549 cells. **d** Cell morphology of β3, D119A β3, and ∆cyto β3 integrin-expressing A549 cells. Scale bar, 50 µm. **e** Western blotting of EMT markers in control, β3, D119A β3, and ∆cyto β3 integrin-expressing A549 cells. **f**, **g** Cell migration (**f**) and invasion (**g**) of the indicated A549 cells. One-way ANOVA and Bonferroni post-hoc test, mean ± s.e.m. of three independent assays conducted in triplicate. The source data are provided in Supplementary Data 1. Unprocessed blot images in (**a**, **c** and **e**) are shown in Supplementary Fig. 6.

Integrin-mediated cell adhesion to ECM activates downstream signaling pathways that contribute to cancer cell proliferation, migration, and invasion^12^. To determine whether αvβ3 integrin-mediated cell adhesion and the subsequent cellular signaling are required for αvβ3 integrin-induced partial EMT, we established A549 cell lines stably expressing two β3 integrin mutants, D119A and ∆cyto β3 integrins. D119A β3 integrin is a mutant that is unable to bind ligands, and ∆cyto β3 integrin is a cytoplasmic deletion mutant that is unable to send β3 integrin signaling within cells (Fig. 5a). Indeed, both D119A and ∆cyto β3 integrins expressing A549 cells significantly reduced cell adhesion to fibronectin via αvβ3 integrin and FAK phosphorylation, compared to the β3-A549 cells (Fig. 5b, c). The β3 integrin mutant-expressing A549 cells showed epithelial morphology, whereas the β3-A549 cells formed an elongated fibroblast-like morphology (Fig. 5d). Furthermore, both mutants lost the ability to induce N-cadherin and vimentin expression (Fig. 5e) and to promote cell migration and invasion (Fig. 5f, g). From these results we conclude that, αvβ3 integrin-mediated cell adhesion and the subsequent signals are required to induce partial EMT, followed by cancer cell migration and invasion.

### TGF-β1 synergistically enhances EMT marker expressions and invasiveness but not motility in αvβ3 integrin expressing lung cancer cells

**Fig. 6:**
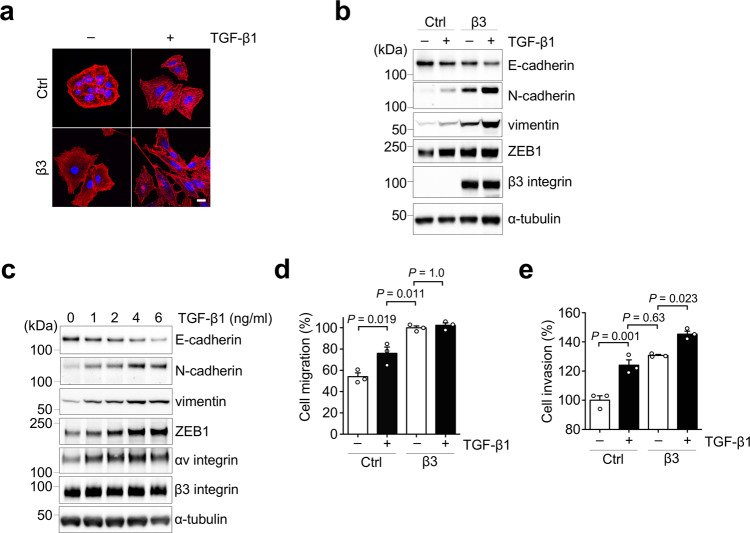
Synergistic effect of αvβ3 integrin and TGF-β1 on EMT induction. **a** F-actin staining (red) of control and β3-A549 cells in the absence or presence of TGF-β1. Images are shown with nuclear stain (blue). Scale bar, 10 µm. **b** Western blotting of EMT markers in control and β3-A549 cells in the absence or presence of 2 ng/ml TGF-β1 for 24 h. **c** Western blotting of EMT markers in β3-A549 cells treated with different concentrations of TGF-β1. **d**, **e** Effect of TGF-β1 treatment on the cell migration (**d**) and invasion (**e**) of control and β3-A549 cells. One-way ANOVA and Bonferroni post-hoc test, mean ± s.e.m. of three independent assays conducted in triplicate. The source data are provided in Supplementary Data 1. Unprocessed blot images in (**b** and **c**) are shown in Supplementary Figs. 7 and 8.

TGF-β is the most thoroughly studied EMT inducer^7^. We therefore examined whether TGF-β1 affects αvβ3 integrin-induced partial EMT. Treatment of β3-A549 cells with TGF-β1 exhibited a more spindle-shaped morphology when compared with non-TGF-β1-treated β3-A549 cells (Fig. 6a). Furthermore, TGF-β1 increased the expression levels of mesenchymal markers such as N-cadherin, vimentin, and ZEB1, and decreased the expression level of epithelial marker E-cadherin in β3-A549 cells (Fig. 6b). The synergistic effect of TGF-β1 on EMT marker changes in β3-A549 cells was observed in a dose-dependent manner (Fig. 6c).

To examine whether TGF-β1 affects the functions of αvβ3 integrin-induced partial EMT, we next conducted cell migration and invasion assays. TGF-β1 treatment increased cell motility and invasiveness in the control A549 cells, whereas in the β3-A549 cells, TGF-β1 treatment promoted cell invasion but not cell migration (Fig. 6d, e). These data suggest that TGF-β1 can generate heterogeneous EMT states with distinct properties by cooperation with αvβ3 integrin.

### αvβ3 Integrin induces partial EMT by a TGF-β-independent mechanism

**Fig. 7:**
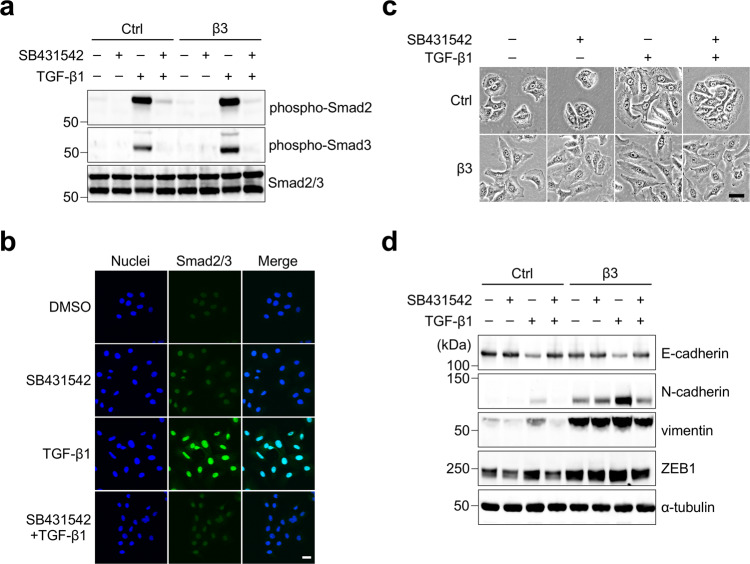
Partial EMT induced by αvβ3 integrin is independent of TGF-β signaling. **a** Western blotting of phospho-Smad2, phospho-Smad3, and Smad2/3 in control and β3-A549 cells pretreated with or without 5 µM SB431542 for 20 min and then treated with 2 ng/ml TGF-β1 for 30 min either separately or in combinations. **b** Anti-Smad2/3 immunofluorescence staining (green) of β3-A549 cells treated with DMSO, 2 µM SB431542, and 4 ng/ml TGF-β1 for 2 days either separately or in combinations. Nuclear staining (blue) was used to detect nuclei and the co-localization of nuclei with Smad2/3 in the merged panel. Scale bar, 25 µm. **c** Cell morphology of control and β3-A549 cells pretreated with or without 5 µM SB431542 for 20 min at room temperature and then treated with 5 ng/ml TGF-β1 for 17 h either separately or in combinations. Scale bar, 40 µm. **d** Western blotting of EMT markers in control and β3-A549 cells pretreated with or without 5 µM SB431542 for 20 min at room temperature and then treated with 5 ng/ml TGF-β1 for 2 days either separately or in combinations. Unprocessed blot images in (**a** and **d**) are shown in Supplementary Fig. 9.

In the current study, since TGF-β1 synergistically promoted EMT progression with αvβ3 integrin, it is possible that αvβ3 integrin-induced partial EMT in a TGF-β-independent fashion. To test this hypothesis, we examined phosphorylation of Smad2 and Smad3, as well as their nuclear translocation, which is induced by activation of TGF-β signaling. We detected no upregulation of the TGF-β-Smad signaling pathway in non-TGF-β1-treated β3-A549 cells (Fig. 7a, b) or in the tumors arising from β3-A549 cells (Supplementary Fig. 2e). In contrast, TGF-β1 treatment induced Smad2/3 phosphorylation (Fig. 7a) and their nuclear translocation (Fig. 7b) in β3-A549 cells. The activation of the TGF-β-Smad signaling pathway was completely abolished by treatment with SB431542, an inhibitor of the type I TGF-β receptor (Fig. 7a, b).

TGF-β1 induced cell morphological change from epithelial to mesenchymal phenotype in the control A549 cells, which was reversed by SB431542 treatment (Fig. 7c). In contrast, the β3-A549 cells kept their fibroblast-like morphology despite the presence of SB431542 (Fig. 7c). The TGF-β1-induced marker changes, elevated expression of N-cadherin, vimentin, and ZEB1, and decreased expression of E-cadherin, were completely abolished by SB431542 treatment in both the control and β3-A549 cells (Fig. 7d). These findings support the notion that partial EMT induction by αvβ3 integrin is independent of the TGF-β signaling pathway.

### miR-200 family diminished αvβ3 integrin-induced partial EMT by suppressing cell surface expression of αvβ3 integrin

**Fig. 8:**
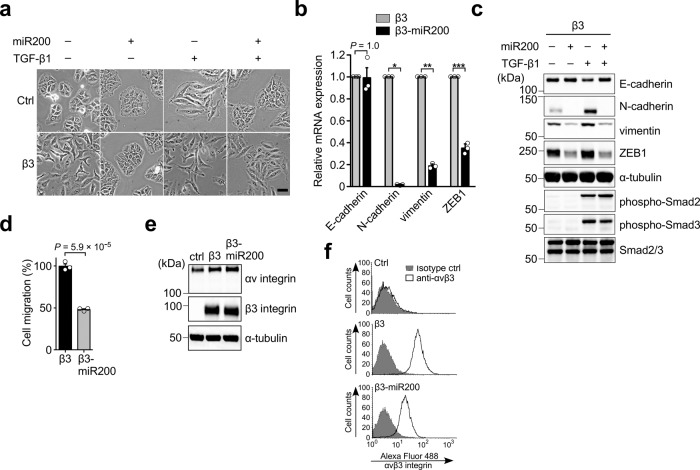
miR-200 family can prevent αvβ3 integrin-mediated partial EMT. **a** Cell morphology of control A549 cells, β3-A549 cells, and control and β3-A549 cells stably expressing the miR-200 family (β3-miR200-A549 cells) with or without 2 ng/ml TGF-β1 treatment for 24 h. Scale bar, 100 µm. **b** Relative mRNA expression levels (qRT-PCR) of EMT markers in β3-A549 and β3-miR200-A549 cells. One-way ANOVA and Bonferroni post-hoc test, mean ± s.e.m. of three independent assays conducted in triplicate. ^*^P = 4.2 × 10^−11^, ^**^P = 7.6 × 10^−10^, ^***^P = 2.1 × 10^−8^. **c** Western blotting of EMT markers in β3-A549 and β3-miR200-A549 cells with or without 2 ng/ml TGF-β1 treatment for 24 h. **d** Effect of the miR-200 family expression on the cell migration of β3-A549 cells. Two-tailed unpaired Student’s *t*-test, mean ± s.e.m. of three independent assays conducted in triplicate. **e** Western blotting analysis of the intracellular expression levels of αv and β3 integrins in control, β3- and β3-miR200-A549 cells. **f** FACS analysis of the cell surface expression of αvβ3 integrin in control, β3-, and β3-miR200-A549 cells. The source data are provided in Supplementary Data 1. Unprocessed blot images in (**c** and **e**) are shown in Supplementary Fig. 10.

The miR-200 family is one of the best-studied EMT regulators^8,27^. Therefore, we tried to examine whether it can affect αvβ3 integrin-induced partial EMT. Enforced expression of the miR-200 family in the β3-A549 (β3-miR200-A549) cells reversed the αvβ3 integrin-induced fibroblast-like morphology to epithelial cobblestone morphology even in the presence of TGF-β1 (Fig. 8a). Consistently, we observed that the β3-miR200-A549 cells showed a decrease in mesenchymal markers, N-cadherin, vimentin, and ZEB1, at both RNA (Fig. 8b) and protein (Fig. 8c) levels compared to the β3-A549 cells. Similar results were also obtained in the presence of TGF-β1 (Fig. 8c). The E-cadherin expression in the β3-A549 cells was suppressed by TGF-β1 treatment, but was restored by the introduction of the miR-200 family gene (Fig. 8c). In contrast, expression of the miR-200 family had no effect on TGF-β1-induced phosphorylation of Smad2 and Smad3 in the β3-A549 cells (Fig. 8c).

To determine the effect of the miR-200 family expression on the function of αvβ3 integrin-induced partial EMT, we performed a transwell migration assay with β3-miR200-A549 cells (Fig. 8d). We found that the miR-200 family expression significantly decreased αvβ3 integrin-induced cell motility.

To investigate the mechanisms by which the miR-200 family reverses αvβ3 integrin-induced partial EMT, we examined the expression of αvβ3 integrin. Intracellular expression levels of αv and β3 integrins were comparable between the β3- and β3-miR200-A549 cells (Fig. 8e). In contrast, FACS analyses revealed that the miR-200 family diminished the cell surface expression of αvβ3 integrin in the β3-A549 cells (Fig. 8f, β3 versus β3-miR200). These data suggest that in lung adenocarcinoma tumors, a decreased level of the miR-200 family might facilitate αvβ3 integrin-mediated partial EMT and cancer progression.

## Discussion

αvβ3 integrin is known to play a pivotal role in lung cancer progression and TGF-β-mediated EMT. Here, we found an unexpected result, where overexpression of αvβ3 integrin in lung cancer cells induced partial EMT in a TGF-β-independent fashion. β3 integrin was significantly increased in lung adenocarcinoma tumor tissues compared to normal lung tissues. The elevated β3 integrin expression was associated with poor survival in lung adenocarcinoma patients. We also demonstrated that αvβ3 integrin-induced partial EMT more efficiently promoted cancer cell migration, invasion, tumorigenesis, stemness, and metastasis, and that TGF-β1 synergistically promoted EMT progression with αvβ3 integrin. The enhanced EMT promoted the invasiveness but not the motility of lung cancer cells compared with the EMT induced by TGF-β1 alone. Furthermore, the miR-200 family abolished the αvβ3 integrin-induced partial EMT by suppressing the cell surface expression of αvβ3 integrin.

It is commonly believed that cancer invasion and metastasis initiate after the loss of E-cadherin, because E-cadherin-mediated cell–cell adhesion prevents cancer cell migration^28^. However, our study demonstrates that αvβ3 integrin-induced partial EMT promotes migration and invasion of cancer cells even with a sufficient amount of E-cadherin expression. This finding is partly supported by several studies reporting that E-cadherin plays important roles in the dissemination, intravasation, and survival of cancer cells^29,30^. Notably, almost half of primary lung adenocarcinomas coexpress vimentin and fibronectin, with high levels of E-cadherin^31,32^. The lower ratios of E-cadherin-to-mesenchymal markers were not associated with poor prognosis in NSCLC patients or aggressiveness in lung adenocarcinoma cell lines^31,33^. In contrast to previous studies^27,34^, our data showed that the increased expression of ZEB1 by αvβ3 integrin had no significant effect on E-cadherin expression [Fig. 7d, ctrl (−/−) versus β3 (−/−)]. Thus, these observations suggest that partial EMT-mediated tumor functions are unpredictable by using the expression patterns of conventional EMT markers such as E-cadherin and ZEB1.

Immunofluorescence-based single cell analyses using anti-E-cadherin and anti-vimentin antibodies demonstrated that partial EMT state in the β3-A549 cells was maintained both in vitro (Fig. 2g) and in vivo (Supplementary Fig. 2c). Recent reports have shown that OVOL1 acts as a critical molecular brake on EMT, and maintains cells in a partial EMT phenotype^11,35,36^. Because OVOL1 was expressed in the β3-A549 cells, it is possible that OVOL1 stabilized the partial EMT phenotype in the β3-A549 cells. Further studies are needed to reveal the partial EMT-associated markers and the mechanisms underlying the stability of partial EMT.

Since miR-200 suppressed cell surface expression levels of αvβ3 integrin but not intracellular expression levels of αv and β3 integrins between the β3- and β3-miR200-A549 cells (Fig. 8e), miR-200 may indirectly alter the cell surface expression levels of αvβ3 integrin through downregulation of the target molecules that affect cell surface expression of αvβ3 integrin. The present study also demonstrated that the miR-200 family suppressed ZEB1 expression in the β3-A549 cells. A similar result has previously been reported, in that ZEB1 knockdown increased cell surface expression of β4 integrin in prostate cancer cells^37^. These observations have led to speculation that the miR-200 family regulates cell surface expression of integrins through ZEB1 regulation.

The findings of the current study demonstrate that αvβ3 integrin-induced partial EMT enhanced cancer cell motility, which was suppressed by miR-200 expression. However, how αvβ3 integrin regulates EMT marker expression, and how the miR-200 family suppresses cell surface expression of αvβ3 integrin, remain unclear. A further comprehensive study is required to understand the role of αvβ3 integrin expression in in vivo tumor functions.

We also found that αvβ3 integrin-induced partial EMT is independent of TGF-β signaling, and has increased cell motility and invasiveness compared with TGF-β1-induced EMT. Notably, reduced expression of type II TGF-β receptor is associated with aggressive tumor behavior^20,38,39^. These observations suggest that TGF-β is not always required to induce the EMT phenotype that is associated with tumor malignancy. Nevertheless, our data show that TGF-β1 downregulates E-cadherin expression in a dose-dependent manner, and promotes αvβ3 integrin functions in β3-A549 cells. Since TGF-β1 treatment selectively downregulates the miR-200 family^27^, TGF-β signaling may promote cell surface expression of αvβ3 integrin in cancer cells. Collectively, these findings suggest that the cooperation of TGF-β and αvβ3 integrin signaling can induce various partial EMT states with distinct tumorigenesis, invasiveness, and metastatic potential. Therefore, a combination of anti-cancer drugs targeting αvβ3 integrin and TGF-β signaling might be more effective than either drug alone.

In conclusion, the results of the present study suggest that αvβ3 integrin is a key driver of partial EMT, and generates tumor diversity. Additionally, we found that TGF-β1 may promote EMT progression synergistically with αvβ3 integrin. Therefore, αvβ3 integrin-induced partial EMT may be a therapeutic target for lung cancer treatments.

## Methods

### Cell culture, transfection, and infection

Human lung cancer cell lines A549 and human embryonic kidney cell lines 293 T were obtained from the RIKEN BRC through the National Bio-Resource Project of the MEXT, Japan. Modified human 293 phoenix cells were a gift from Dr. M Peter Marinkovich (Stanford University, Stanford, CA). These cells were maintained in Dulbecco’s modified Eagle’s medium (DMEM; #043-30085, WAKO) and were supplemented with 10% fetal bovine serum (FBS), penicillin, and streptomycin sulfate (#168-23191, WAKO). Human lung cancer cell lines, H358, H460, and H1975 cells, were purchased from the American Type Culture Collection (ATCC), and were grown in RPMI-1640 (#189-02025, WAKO), supplemented with 10% FBS, 2.5 g/l D (+)-glucose, 1 mM sodium pyruvate, and 10 mM HEPES. Retroviruses were produced from 293 phoenix cells transfected with Lipofectamine LTX & Plus reagent (#15338-030, Life Technologies) and LZRS blast retroviral vectors. The cells were then selected with 5 µg/ml puromycin (#P8833, Sigma-Aldrich). Lentiviruses were produced from 293T cells using pLKO.1 vectors and the Trans-Lentiviral shRNA Packaging kit (#TLP5912, GE Healthcare) according to the manufacturer’s instructions. Viral supernatant was passed through a 0.45 µm filter and stored at −80 °C until use. Infection was performed as described previously^40^. One day before infection, 4 × 10^5^ cells in 3 ml growth media were plated in a six-well plate (#353846, BD Falcon). After incubation with 5 µg/ml polybrene (#10768-9, Sigma-Aldrich) for 15 min, the media were exchanged to viral supernatant, and another 5 µg/ml polybrene was added. After centrifuging the plate at 1200 rpm for 1 h at 32 °C using a Hitachi CR22N centrifuge machine, the viral supernatant was replaced with fresh growth medium. To establish a cell line, cells were selected with 10 µg/ml blasticidin S (#203350, Calbiochem) for retroviral infection and 5 µg/ml puromycin for lentiviral infection. For all experiments, all cells were cultured less than 6 months after purchasing or receiving them, and were tested for mycoplasma contamination. Cell morphology was observed under an IX71 phase contrast microscope (Olympus), and photographs were taken.

### Immunohistochemistry analysis

Human lung adenocarcinoma tissue arrays (#LC641 and #LC10013c) were purchased from US Biomax. After baking for 1 h at 60 °C, tissue slides were immersed in Tris-EDTA buffer (10 mM Tris-base, 1 mM EDTA, 0.05% Tween 20, pH9.0) and subjected to microwave treatment for 15 min for antigen retrieval. The tissue samples were deparaffinized, rehydrated, and immersed in 0.3% H_2_O_2_ in methanol for 20 min at room temperature to inactivate endogenous peroxidase. Samples were blocked with 5% skim milk/PBS for 1 h at room temperature, incubated with anti-β3 integrin primary antibody (#ab179473, Abcam, 1:800) in 5% skim milk/PBS overnight at 4 °C, and rinsed three times in PBS for 5 min. The sections were incubated with peroxidase-labeled secondary antibody [Histofine simple stain MAX-PO (MULTI) kit, #424152, Nichirei Corp.] for 30 min at room temperature. After washing three times with PBS for 5 min, peroxidase was visualized using Histofine DAB substrate kit (#425011, Nichirei Corp.), and slides were counterstained with Meyer’s hematoxylin solution (#131-09665, WAKO). Images were obtained using a Nanozoomer-SQ (#C13140-L04, Hamamatsu) and analyzed using NDPview2 software. Histological scores were determined based on the β3 integrin staining intensity in a blinded fashion by two independent investigators. In addition to carcinoma cells, some host-reactive stromal cells were also stained, but these have been excluded from the following analyses.

### Reagents, plasmids, and shRNAs

The TGF-β1 receptor inhibitor SB431542 was from WAKO (#192-16541). Recombinant human TGF-β1 was from Peprotech (#100-21). Retroviral LZRS and lacZ-LZRS blast expression vectors were a gift from Dr. M Peter Marinkovich (Stanford University, Stanford, CA). LZRS blast expression vector encoding β3 integrin was prepared as previously described^41^. All mutants were generated by oligonucleotide site-directed mutagenesis. A retroviral lacZ-LZRS blast expression vector was used as a control. All cDNA sequences were verified by DNA sequencing. pLKO.1 lentiviral plasmids encoding shRNAs targeting human β3 integrin (clones TRCN0000003234 and TRCN0000003236) and control shRNA (#RHS4459) were from Dharmacon. Lentiviral vector pLenti4.1 Ex miR200b-200a-429 was a gift from Greg Goodall (Addgene #35533)^27^.

### RNA extraction and quantitative RT-PCR

Total RNA was extracted using the NucleoSpin RNA Plus kit (#740984.50, Macherey-Nagel) and reverse-transcribed with the PrimeScript RT Master Mix kit (RR036A, Takara). Quantitative PCR (qPCR) was performed with the PrimeTime Gene Expression Master Mix (#1055770, IDT) and primers from PrimeTime Mini qPCR Assay system (IDT) on a StepOne PCR machine (Thermo Fisher Scientific) according to the manufacturer’s instructions. Data were collected and analyzed using StepOne software. All mRNA quantification data were normalized to HPRT1.

Primer sequences used:

E-cadherin forward, 5ʹ-CTGAGGATGGTGTAAGCGATG-3ʹ;

E-cadherin reverse, 5ʹ-GTCTGTCATGGAAGGTGCTC-3ʹ;

N-cadherin forward, 5ʹ-CATACCACAAACATCAGCACAAG-3ʹ;

N-cadherin reverse, 5ʹ-GTTTGCCAGTGTGACTCCA-3ʹ;

vimentin forward, 5ʹ-GTGAATCCAGATTAGTTTCCCTCA-3ʹ;

vimentin reverse, 5ʹ-CAAGACCTGCTCAATGTTAAGATG-3ʹ;

ZEB1 forward, 5ʹ-GGCATACACCTACTCAACTACG-3ʹ;

ZEB1 reverse, 5ʹ-CCTTCTGAGCTAGTATCTTGTCTTTC-3ʹ;

OVOL1 forward, 5ʹ-CAAGAGACACGTCCGAACTC-3ʹ;

OVOL1 reverse, 5ʹ-CATGGATCTTCTTGAGGTGAGA-3ʹ;

HPRT1 forward, 5ʹ-GCGATGTCAATAGGACTCCAG-3ʹ;

HPRT1 reverse, 5ʹ-TTGTTGTAGGATATGCCCTTGA-3ʹ.

### Western blotting

For preparing cell lysates, the cells were washed twice with cold PBS and then lysed with a lysis buffer [1% Triton X-100, 20 mM Tris-HCl (pH 7.4), 150 mM NaCl, 5 mM EDTA] containing a protease inhibitor cocktail (#25955-24; Nacalai Tesque) and a phosphatase inhibitor cocktail (#07575-51; Nacalai Tesque). After incubation for 20 min on ice, the cell lysates were clarified by centrifugation at 15,000 rpm for 20 min at 4 °C, and the resultant supernatant was used as a cell lysate sample. The protein concentration of the cell lysate sample was determined using a protein assay kit (#29449-44, Nacalai Tesque). For western blotting, proteins resolved by SDS-PAGE under reducing condition were transferred to nitrocellulose membranes. The membranes were blocked with 5% skim milk in TBST for 1 h at room temperature, washed three times with TBST for 5 min, and probed with primary antibodies for 1 h at room temperature or overnight at 4 °C. Primary antibodies against the following proteins were used: mouse monoclonal antibodies specific for E-cadherin (clone 36, #610182, BD Transduction Laboratories, 1:1000), N-cadherin (clone 21/CD51, #610920, BD Transduction Laboratories, 1:1000), αv integrin (clone 21, #611012, BD Transduction Laboratories, 1:1000), β3 integrin (clone 1, #611140, BD Transduction Laboratories, 1:1000), vimentin (clone V9, #sc-6020, Santa Cruz Biotechnology, 1:1000), α-tubulin (clone DM 1 A, #T9026, SIGMA, 1:5000), FAK (clone 77, #610087, BD Transduction Laboratories, 1:1000), phospho-FAK (clone 18, #611806, BD Transduction Laboratories, 1:1000), rabbit monoclonal antibodies against ZEB1 (clone D80D3, #3396, Cell Signaling Technology, 1:1000), Smad2/3 (clone D7G7, #8685, Cell Signaling Technology, 1:1000), phospho-Smad2 (clone 138D4, #3108, Cell Signaling Technology, 1:1000), and phospho-Smad3 (clone C25A9, #9520, Cell Signaling Technology, 1:1000), rabbit polyclonal antibodies against ZO-1 (#GTX108592, Gene Tex, 1:1000), and OVOL-1 (#14082-1-AP, Proteintech, 1:1000). Membranes were washed three times with TBST for 5 min and incubated with horseradish peroxidase-conjugated anti-mouse IgG (#7076, Cell Signaling Technology, 1:5000) or anti-rabbit IgG (#W401B, Promega, 1:5000) antibodies for 1 h at room temperature. After washing three times with TBST for 5 min, immunoreactive bands were detected using a Trident femto-ECL reagent (#GTX14698, GeneTex) and imaged using Imager and Image Saver software (#AE-9300H-CP, ATTO). For membrane stripping, the membranes were washed twice with TBST for 5 min. After incubating the prewashed membranes on an orbital shaker with stripping solution (#193-16375, WAKO) for 10 min at room temperature, the membranes were washed three times with TBST for 5 min, and reblocked with 5% skim milk in TBST.

### Fluorescent microscopy

The cells were briefly washed with PBS, fixed with 4% paraformaldehyde/PBS for 10 min, washed three times with PBS for 5 min, permeabilized with 0.5% Triton X-100/PBS for 10 min, and blocked with 2% BSA/TBS for 1 h at room temperature. For the frozen section, the tissues were fixed with acetone for 10 min. The fixed cells were stained with mouse monoclonal antibodies against αvβ3 integrin (clone LM609, #MAB1976, Merck Millipore, 1:100) and E-cadherin (clone HECD1, #M106, Takara, 1:2000), a goat polyclonal antibody against vimentin (clone C-20, #sc-7557, Santa Cruz Biotechnology, 1:100), a rat monoclonal antibody against CD31 (clone MEC13.3, #553370, BD Transduction Laboratories, 1:100), or a rabbit monoclonal antibody against Smad2/3 (clone D7G7, #8685, Cell Signaling Technology, 1:100) for 1 h at room temperature. After washing the cells three times with PBS, bound antibodies were detected with Alexa Fluor 488-conjugated goat secondary anti-mouse IgG and anti-rabbit IgG antibodies (#A11029, Invitrogen, 1:200; #A11034, Invitrogen, 1:500), an Alexa Fluor 546-conjugated donkey secondary anti-goat IgG antibody (#A11056, Invitrogen, 1:500), or an Alexa Fluor 546-conjugated goat secondary anti-rat IgG antibody (#A11081, Invitrogen, 1:500). To detect the nuclei, the cells were stained with NucBlue fixed cell stain readyprobes reagent (#R37606, Molecular probes) or Hoechst 33342 (#382065, Millipore, 0.1 µg/ml). For F-actin staining, the cells were incubated with Alexa Fluor 546-conjugated phalloidin (Invitrogen, #A22283, 1:40). After washing the cells three times with PBS for 5 min, the cells were mounted using fluorescent mounting medium (#S3023, Dako), and fluorescence images were obtained using an A1 confocal microscope (Nikon) or an IX71 fluorescence microscope (Olympus).

### Flow cytometry analysis

To analyze cell surface expression of αvβ3 integrin, the cells were detached from a dish using 0.25% trypsin/PBS with 1 mM EDTA. After quenching trypsinization with a medium that contained 10% FBS, the cells were washed twice with PBS that contained 1 mM EDTA (PBS/EDTA), and then suspended in PBS/EDTA. The cells were then incubated with a mouse monoclonal antibody against αvβ3 integrin (clone 23C6, #304402, BioLegend 1:100) on ice for 30 min. After washing once with PBS/EDTA, the cells were incubated with an Alexa Fluor 488-conjugated goat secondary anti-mouse IgG antibody (1:500). After incubation on ice for 15 min, the cells were washed three times with PBS/EDTA, and then analyzed by flow cytometry using FACSCalibur and CellQuest software (BD Biosciences). At least 10,000 events were analyzed for each sample.

### Cell migration and invasion assays

Cell migration and invasion assays were performed using a 24-well chemotaxis chamber (Cell culture companion plates #353504 and 8.0-µm inserts #352097, BD Transduction Laboratories) as previously described^40^. For the invasion assay, each well was coated with 100 µl of 1.6 mg/ml Matrigel (#354234, BD Transduction Laboratories). The cells (1 × 10^5^ cells in 200 µl serum-free medium) were inoculated into the upper insert, and 750 µl of growth medium was placed in the lower chamber as a chemo-attractant. For the inhibition assay, the cells were incubated with anti-αvβ3 integrin function blocking antibody (23C6, 1:100) for 20 min at room temperature before plating. After incubation for 6 h at 37 °C in a 5% CO_2_ incubator, the cells were briefly washed twice with PBS, fixed with 4% paraformaldehyde for 10 min and stained with 0.5% crystal violet in 20% methanol. The cells on the upper side of the membrane and the Matrigel were removed with cotton swabs. Three randomly chosen fields were photographed using an IX71 phase contrast microscope (Olympus), and the migrated cells were counted.

### Cell proliferation assay

The cells were plated at a density of 8 or 8.3 × 10^4^ cells/35 mm dish in 2 ml growth medium, and incubated at 37 °C in a 5% CO_2_ incubator. After washing twice with PBS, the grown cells were harvested with 0.25% trypsin/PBS with 1 mM EDTA, and the cell numbers were counted using a hemocytometer.

### Cell adhesion assay

The cell adhesion assays were performed as previously described^40^. The wells of 96-well plates (#3590, Corning) were coated with 50 µl of 1 µg/ml human plasma fibronectin (FC010, Millipore) overnight at 4 °C and then blocked with 1% BSA/PBS for 1 h at 37 °C in a 5% CO_2_ incubator. A total of 5 × 10^4^ cells in 100 µl serum-free medium pretreated with or without anti-αvβ3 integrin function blocking antibody (23C6, 1:100) for 20 min at room temperature were inoculated onto the fibronectin-coated wells and incubated for 1 h at 37 °C in a 5% CO_2_ incubator. After removing the non-adherent cells with vigorous shaking, the adherent cells were fixed with 5% (w/v) glutaraldehyde/PBS for 10 min and stained with 0.5% crystal violet in 20% (v/v) methanol for 5 min. After washing the plates with tap water, the dye was extracted with 0.1 M sodium citrate in 50% (v/v) methanol for 30 min, and measured for absorbance at 595 nm using a microplate reader (BioRad, Model 680).

### Kaplan-Meier analysis of overall survival

The Kaplan-Meier analysis of overall survival was generated using the online source http://kmplot.com/analysis.

### In silico co-expression analysis in TCGA datasets

The co-expression between β3 integrin and αv integrin, vimentin, fibronectin, ZEB1, ZEB2, E-cadherin, OVOL1, ZO-1 in patients with lung adenocarcinoma was analyzed using RNA-Seq data (TCGA, https://cancergenome.nih.gov, Firehose Legacy, *n* = 517) and shown in graphs using cBioPortal (https://www.cbioportal.org).

### In vivo tumorigenicity and metastasis assays

All animal studies were performed in accordance with protocols approved by Fukushima Medical University Animal Care and Use Committee. For the tumorigenicity assay, control and β3-A549 cells (1 × 10^6^ cells per 100 µl PBS) were subcutaneously injected along with 100 µl Matrigel into 6-week-old female nude mice (BALB/c nu/nu, SLC). Primary tumor growth was measured once a week with a caliper, and the volume was calculated using the formula *D* (long diameter) × *d* (short diameter)^2^ × 1/2. To assess in vivo tumor cell proliferation, frozen sections of primary tumors (5 µm) were fixed with acetone for 10 min, washed with PBS for 5 min, and blocked with 2% BSA/PBS for 1 h at room temperature. Proliferating cells were stained with a mouse monoclonal antibody against Ki67 (#610968, BD Transduction Laboratories, 1:100). After washing the cells three times with PBS, bound antibodies were detected with an Alexa Fluor 488-conjugated goat secondary anti-mouse IgG antibody (#A11029, Invitrogen, 1:300). To detect the nuclei, the cells were stained with Hoechst 33342 (#382065, Millipore, 0.1 µg/ml). After washing the cells three times with PBS for 5 min, the cells were mounted using fluorescent mounting medium (#0100-01, Southern Biotech). Fluorescence images chosen from three random fields were obtained using an IX71 fluorescent microscope (Olympus). Proliferation was quantified as the ratio of Ki67 staining-to-total nuclear staining. For the metastasis assay, control and β3-A549 cells (1 × 10^6^ cells per 100 µl PBS) were injected into the tail vein of 7-week-old female nude mice. The mice were sacrificed at 7 weeks after injection, and the lungs were removed and fixed in 4% paraformaldehyde/PBS. The paraformaldehyde-fixed tissues were embedded in paraffin, sectioned, and stained with Mayer’s hematoxylin and eosin (HE). Images were obtained using a Nanozoomer-SQ (#C13140-L04, Hamamatsu) and analyzed using NDPview2 software. For the evaluating metastasis, the tumor nodules on the lung section were counted. In all animal experiments, mice were allocated randomly to the experimental and control groups.

### In vitro sphere formation and in vivo tumor initiation assays

For the in vitro sphere assay, control and β3-A549 cells were plated at a density of 1 × 10^5^ cells/96-well ultra-low attachment microplate (#3474, Corning) in 100 µl serum-free DMEM containing B-27 supplement (#17504, Invitrogen, 1:50), 20 ng/ml human epidermal growth factor (#E9644, Sigma), 20 ng/ml human basic fibroblast growth factor (#064-04541, WAKO), and penicillin/streptomycin sulfate. Colonies with a diameter of more than 100 µm were counted after incubation for 4 days at 37 °C in a 5% CO_2_ incubator. For the in vivo tumor initiation assay, control and β3-A549 cells in 100 µl PBS were subcutaneously injected, along with 100 µl Matrigel, into 6-week-old female nude mice (BALB/c nu/nu, SLC). Tumor incidence was observed at 4 weeks after injection.

### Statistics and reproducibility

Results were given as mean ± s.e.m. and were representative of at least three independent experiments for all studies except for the experiments shown in Figs. 1b, 3g–k, and 4b, which were performed once. Statistical comparisons were calculated between two groups using unpaired two-tailed Student’s *t*-test, and among the groups using one-way ANOVA followed by a Bonferroni post-hoc test, with GraphPad Prism Version 5.0a and SPSS statistics 26 software. A *P* value of <0.05 was considered to be statistically significant. Western blotting and micrograph results shown are representative images of three independent experiments, with similar results. No statistical test was used to determine sample size.

### Reporting summary

Further information on research design is available in the Nature Research Reporting Summary linked to this article.

## Supplementary information

Supplementary Information

Description of Additional Supplementary Files

Supplementary Data 1

Reporting Summary

## Data Availability

All data supporting the findings in this study are included in this published article and its supplementary information files. Source data are available in Supplementary Data 1. Any additional information may be available from the corresponding author upon request.
