# Supplementary material for: αvβ3 Integrin induces partial EMT independent of TGF-β signaling
Source: Commun Biol. 2021 Apr 21;4:490. doi: 10.1038/s42003-021-02003-6 (PMC8060333; doi:10.1038/s42003-021-02003-6)
Supplement: Supplementary file 1 — Supplementary Information [file 42003_2021_2003_MOESM1_ESM.pdf]

Supplementary Information

αvβ3 integrin induces partial EMT independent of TGF-β signaling

Yoshinobu Kariya[1,#,*], Midori Oyama[1,#], Takato Suzuki[2,3], Yukiko Kariya[1]

[1]Department of Biochemistry, [2]Laboratory Animal Research Center, Fukushima Medical University School of Medicine, [3]Department of Clinical Laboratory Medicine, Fukushima Medical University Hospital,
1 Hikarigaoka, Fukushima City, Fukushima 960-1295, Japan

[#]Equal contribution

***Corresponding Author:**
Yoshinobu Kariya, Ph.D.
Department of Biochemistry, Fukushima Medical University School of Medicine,
1 Hikarigaoka, Fukushima City, Fukushima 960-1295, Japan.
Phone: +81-24-547-1144; Fax: +81-24-548-8641
E-mail: kariya@fmu.ac.jp

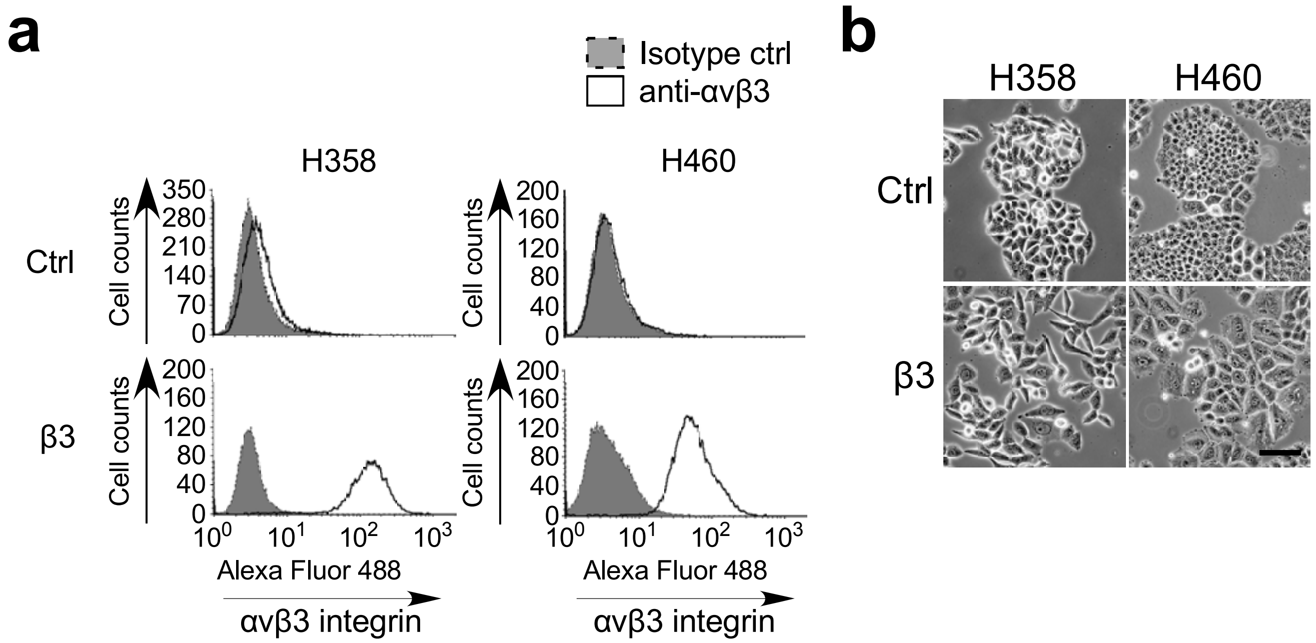

Supplementary Figure 1. Cell morphological changes of H358 and H460 cells by αvβ3 integrin expression. a FACS analysis of the cell surface expression of αvβ3 integrin in control or β3 integrin expressing H358 and H460 cells. **b** Cell morphology of control or β3 integrin expressing H358 and H460 cells. Scale bar, 100 µm.

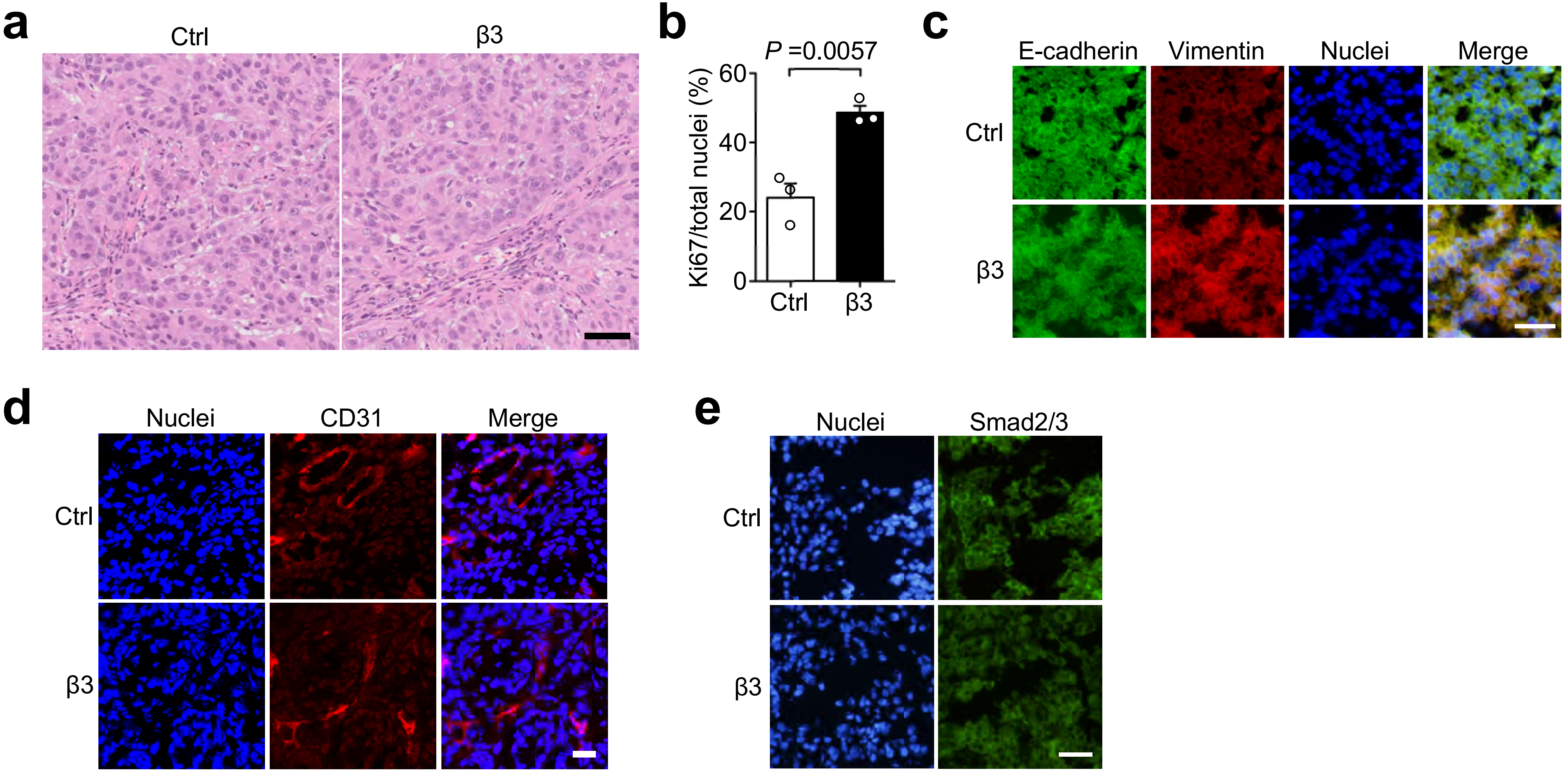

Supplementary Figure 2. Histological characterization of tumors arising from grafting of β3-A549 cells.
a Representative pictures from H&E-stained tumors arising from control and β3-A549 cells. Scale bar, 50 μm.
b *In vivo* cell proliferation assay in tumors arising from control and β3-A549 cells. Two-tailed unpaired Student's t-test, mean ± s.e.m., n = 3 per group. **c** Immunofluorescence-based single cell analysis using anti-E-cadherin (green) and vimentin (red) antibodies in tumors arising from control and β3-A549 cells. n = 3 per group. Scale bar, 50 μm. **d, e** CD31 (**d**, red) and Smad2/3 (**e**, green) immunofluorescence staining in tumors arising from control and β3-A549 cells. n = 3 per group. Scale bars, 50 μm. Nuclear staining was used to detect nuclei (blue). Source data for **b** are available in Supplementary Data 1.

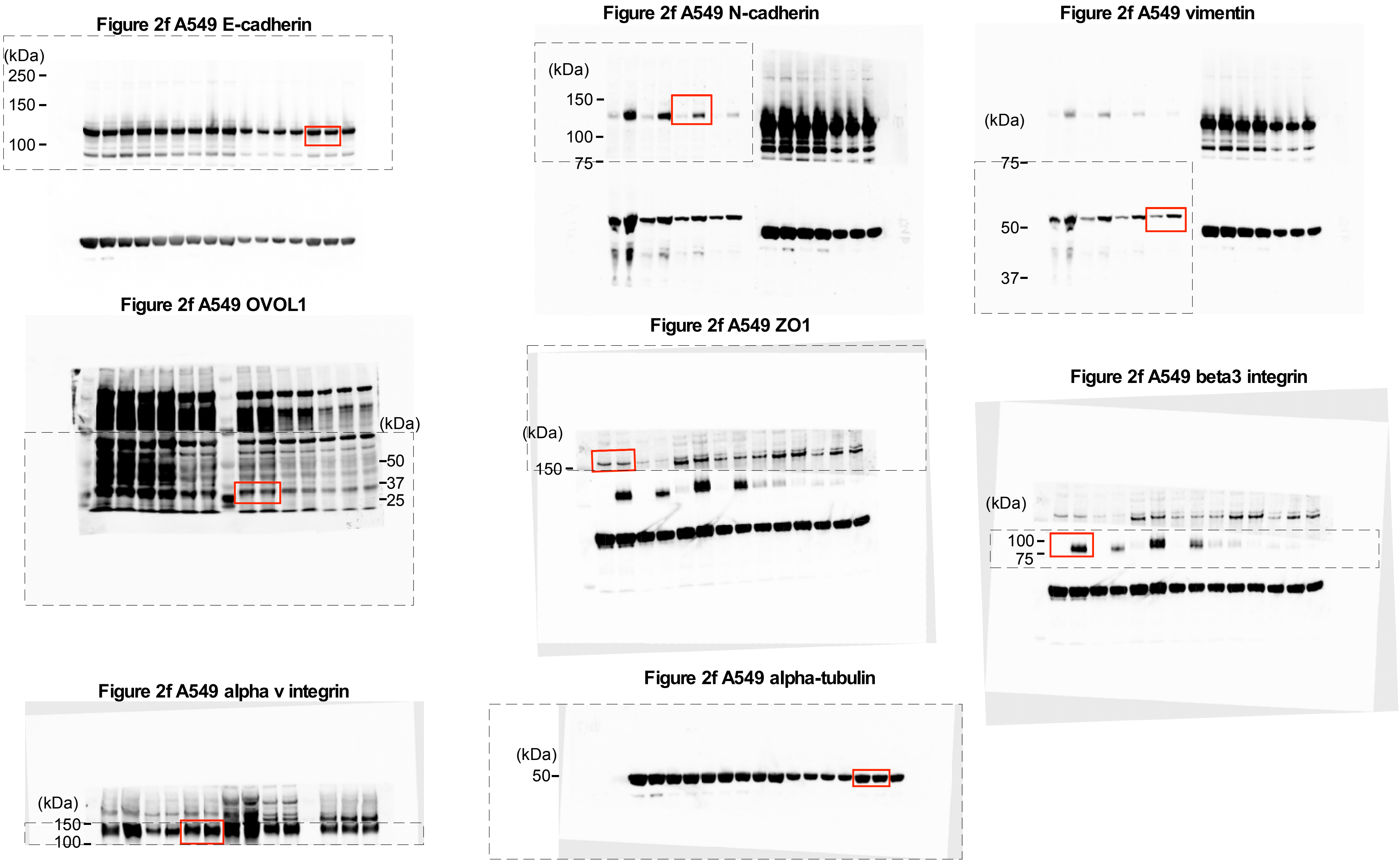

Supplementary Figure 3. Unprocessed blot images in Fig. 2f. Dotted squares indicate blotting membranes. Red squares indicate cropped sections.

Figure 2f H358 E-cadherin

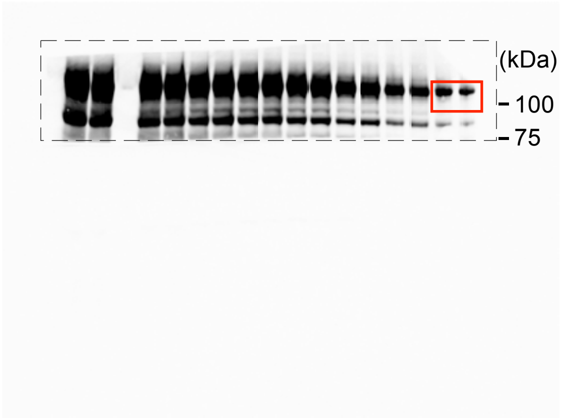

Figure 2f H358 N-cadherin

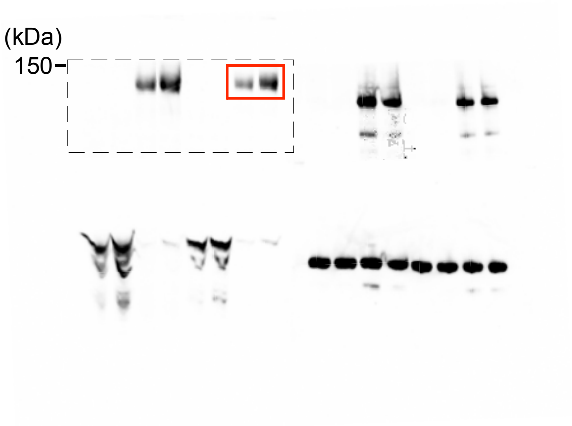

Figure 2f H358 vimentin

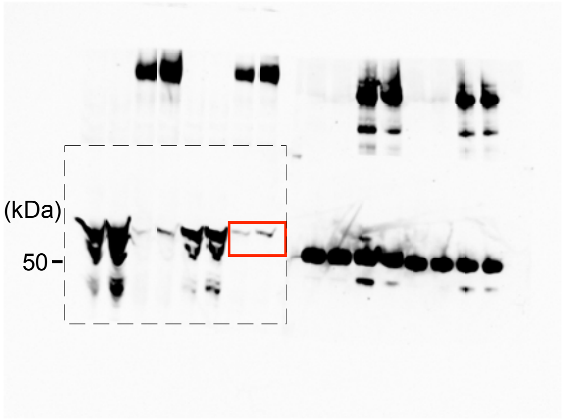

Figure 2f H358 OVOL1

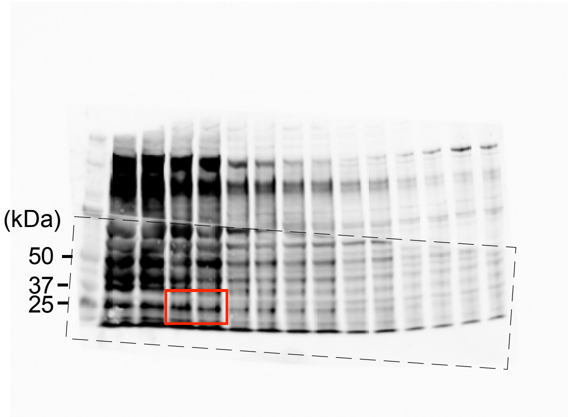

Figure 2f H358 ZO1&beta3 integrin

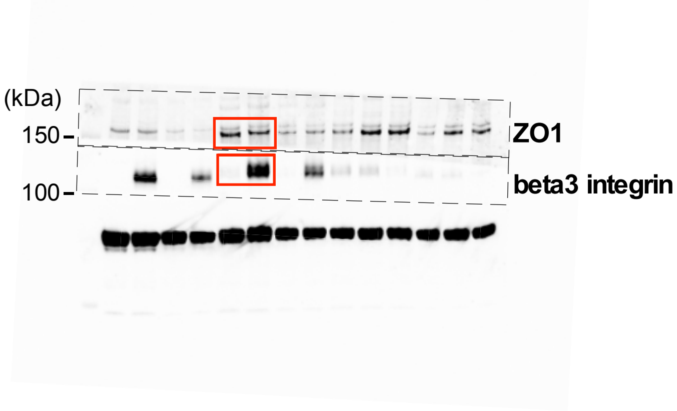

Figure 2f H358 alpha v integrin

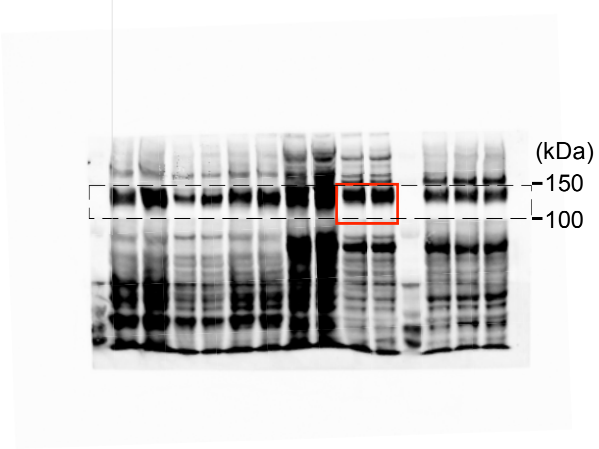

Figure 2f H358 alpha-tubulin

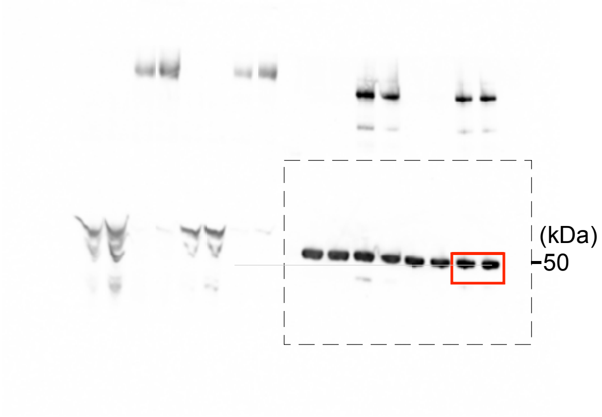

Supplementary Figure 4. Unprocessed blot images in Fig. 2f. Dotted squares indicate blotting membranes. Red squares indicate cropped sections.

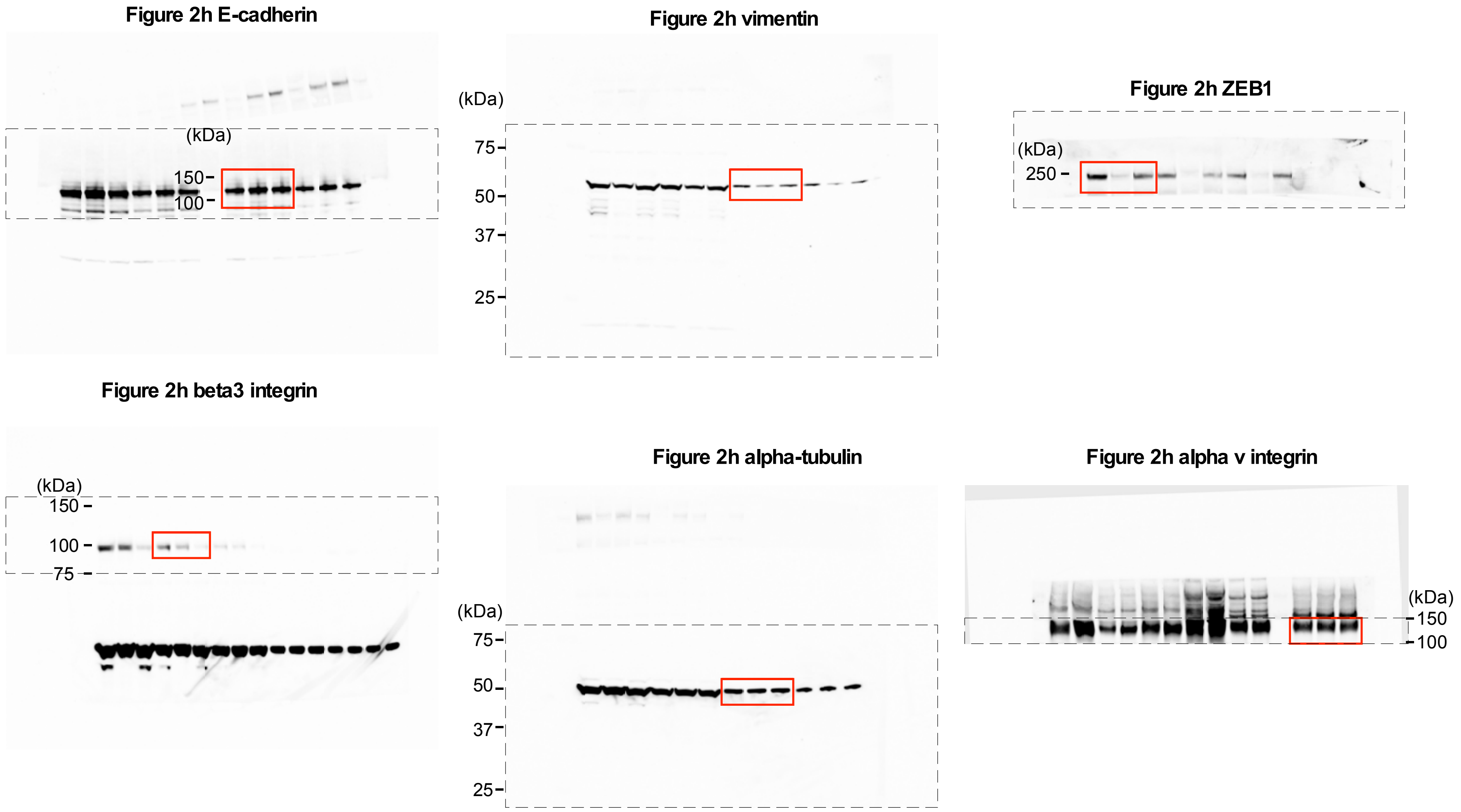

Supplementary Figure 5. Unprocessed blot images in Fig. 2h. Dotted squares indicate blotting membranes. Red squares indicate cropped sections.

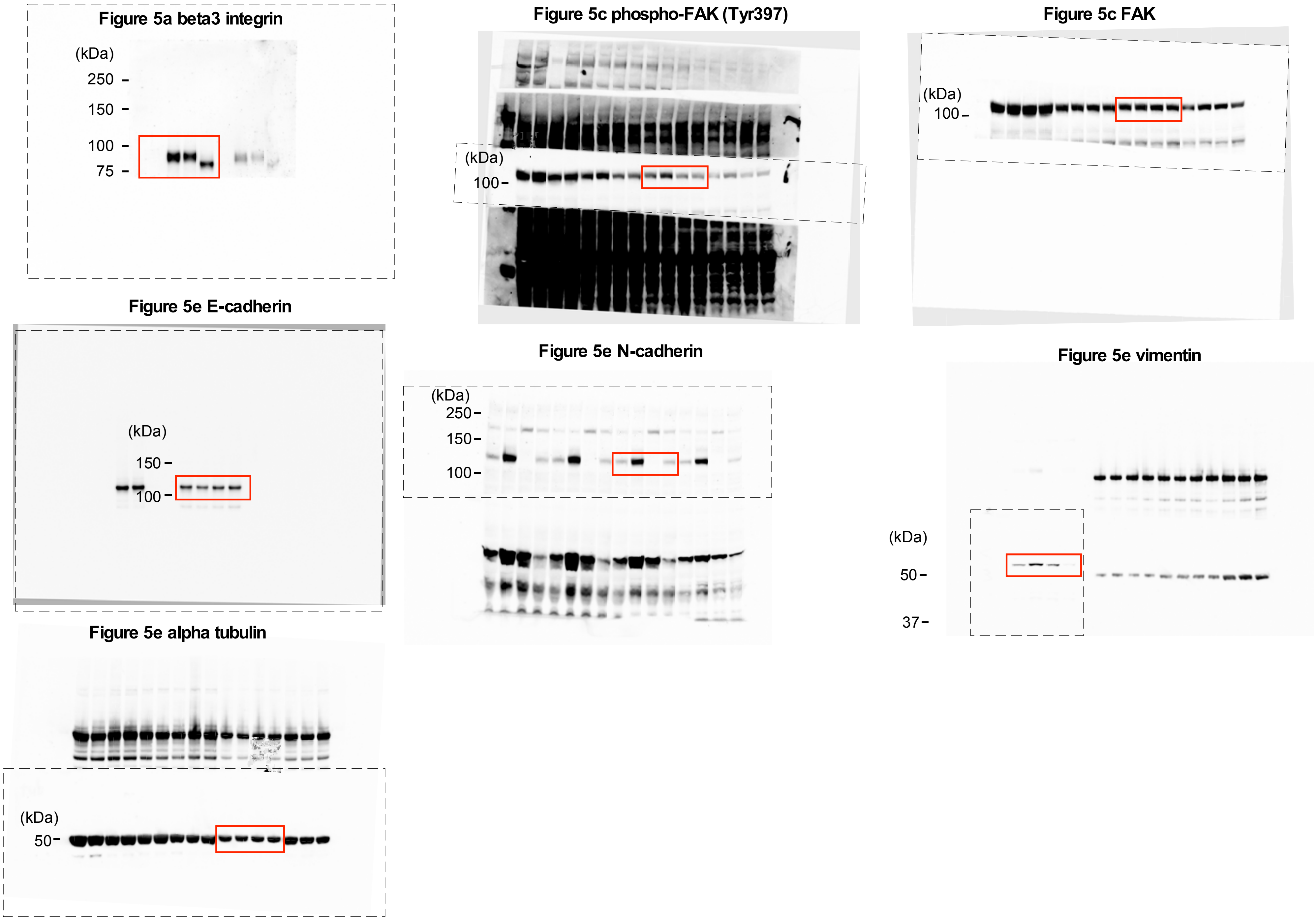

Supplementary Figure 6. Unprocessed blot images in Figs. 5a, c, e. Dotted squares indicate blotting membranes. Red squares indicate cropped sections.

Figure 6b E-cadherin

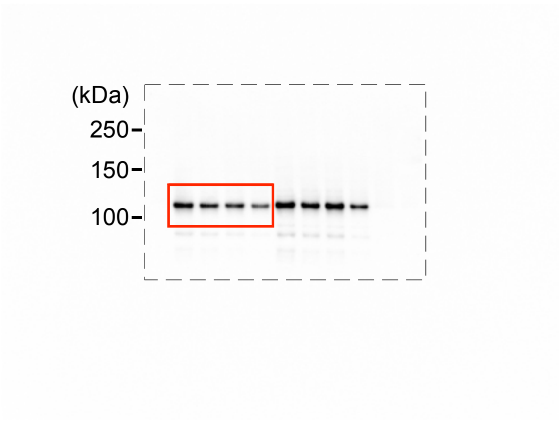

Figure 6b N-cadherin

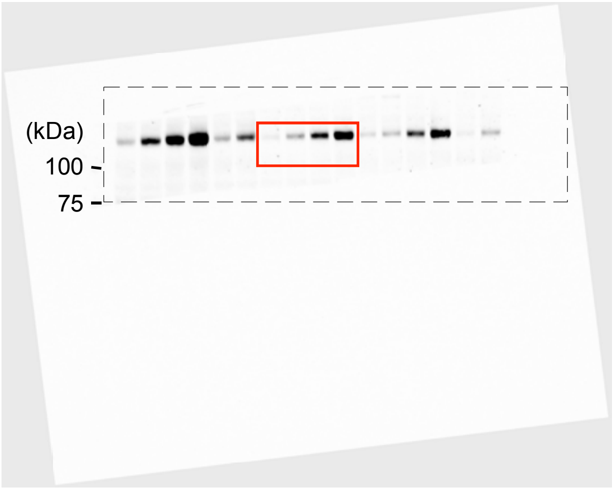

Figure 6b vimentin

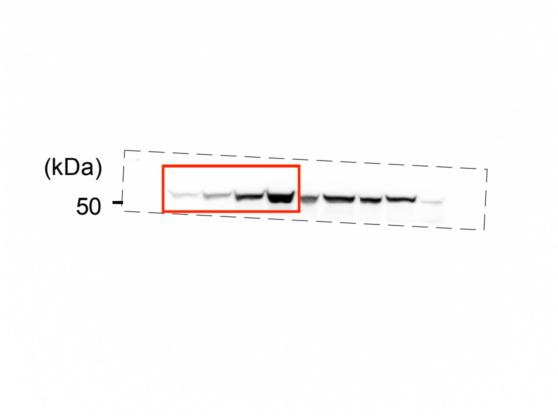

Figure 6b ZEB1

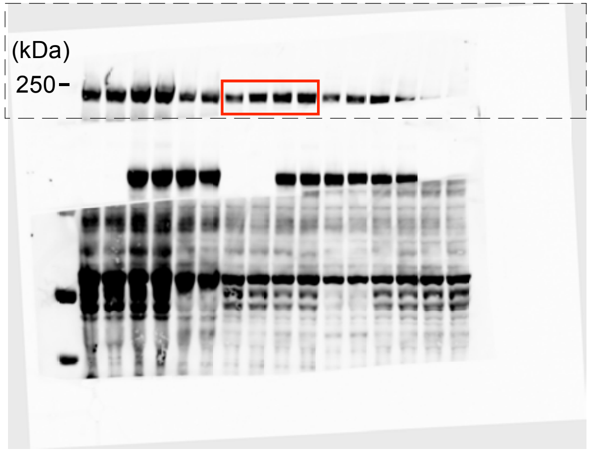

Figure 6b beta3 integrin

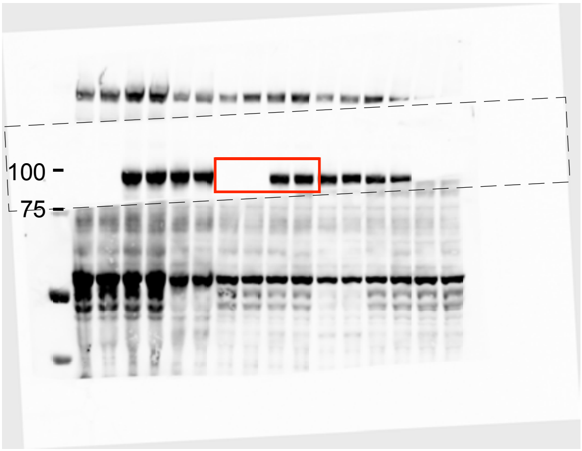

Figure 6b alpha-tubulin

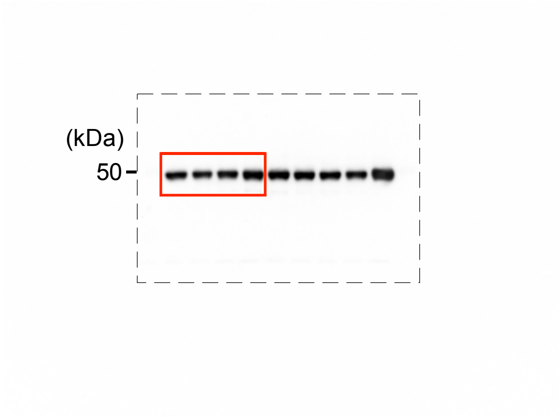

Supplementary Figure 7. Unprocessed blot images in Fig. 6b. Dotted squares indicate blotting membranes. Red squares indicate cropped sections.

Figure 6c E-cadherin

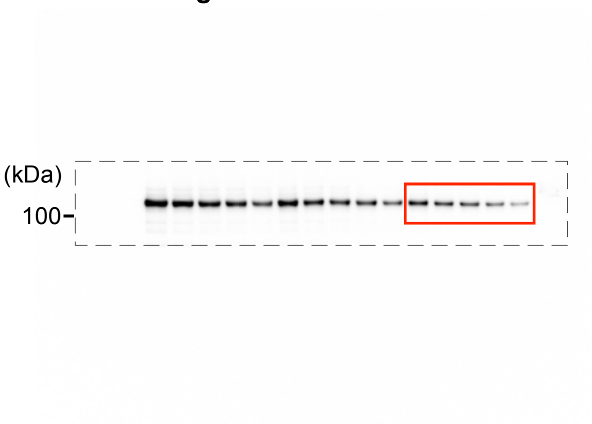

Figure 6c N-cadherin

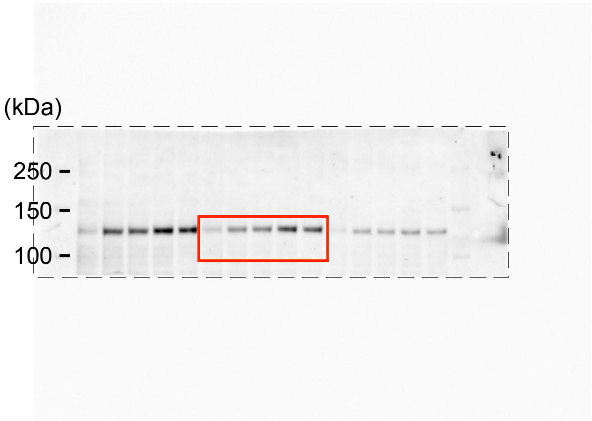

Figure 6c vimentin

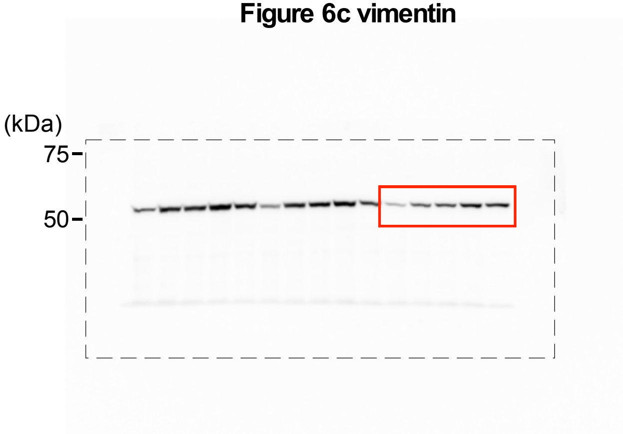

Figure 6c beta3 integrin

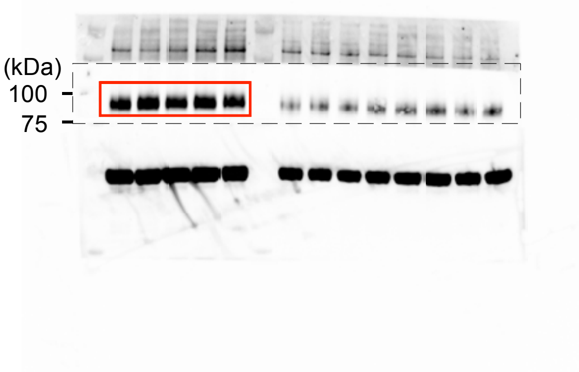

Figure 6c ZEB1

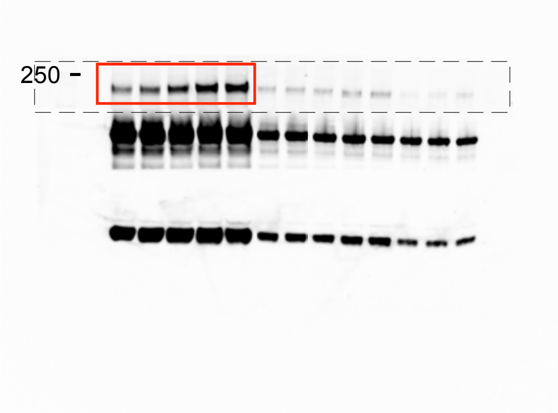

Figure 6c alpha v integrin

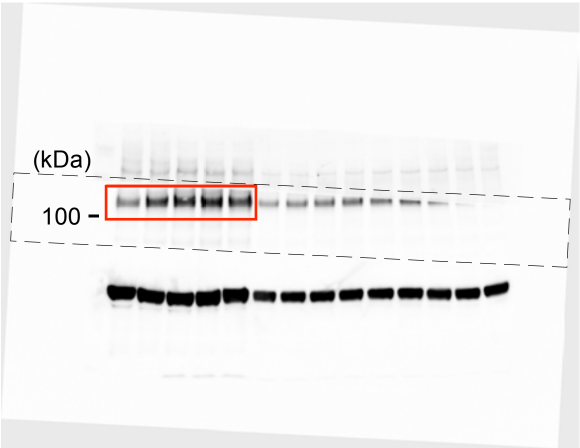

Figure 6c alpha-tubulin

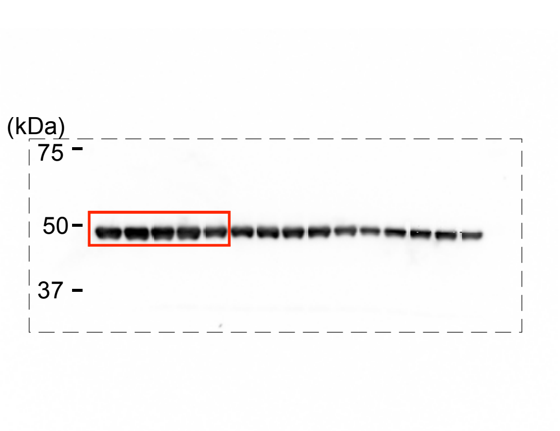

Supplementary Figure 8. Unprocessed blot images in Fig. 6c. Dotted squares indicate blotting membranes. Red squares indicate cropped sections.

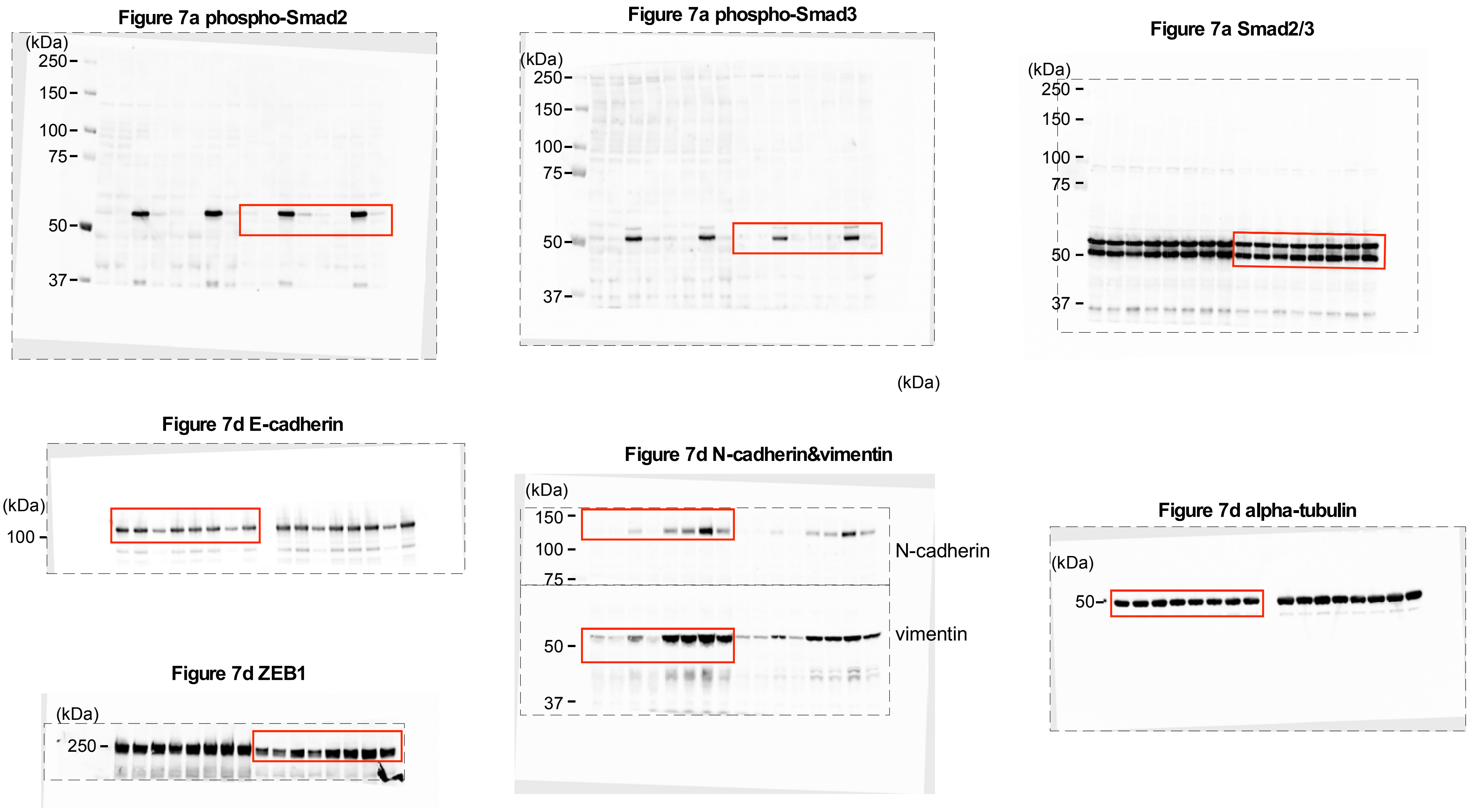

Supplementary Figure 9. Unprocessed blot images in Figs. 7a, d. Dotted squares indicate blotting membranes. Red squares indicate cropped sections.

Figure 8c E-cadherin

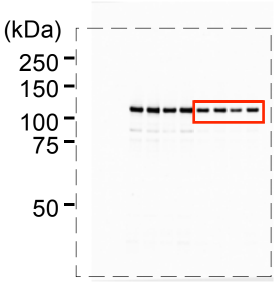

Figure 8c N-cadherin

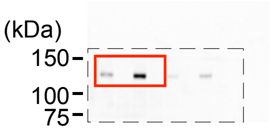

Figure 8c vimentin

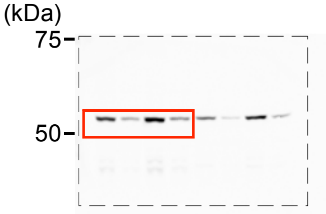

Figure 8c ZEB1

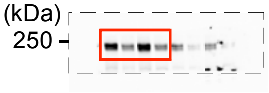

Figure 8c alpha-tubulin

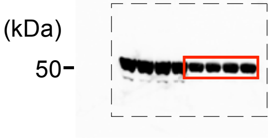

Figure 8c phospho-Smad2

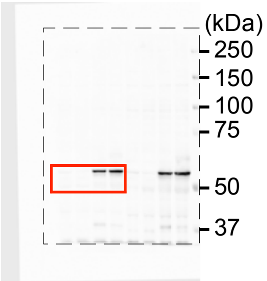

Figure 8c phospho-Smad3

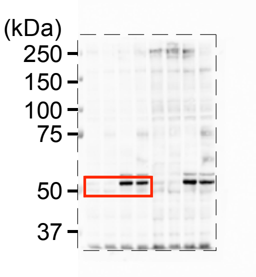

Figure 8c Smad2/3

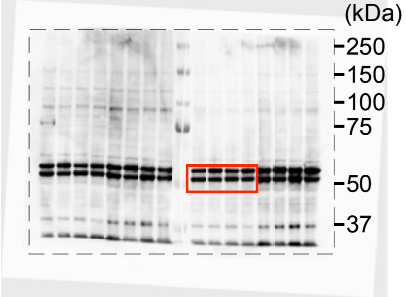

Figure 8e alpha v

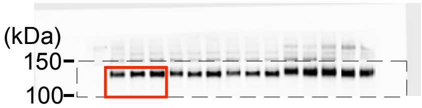

Figure 8e beta3 integrin

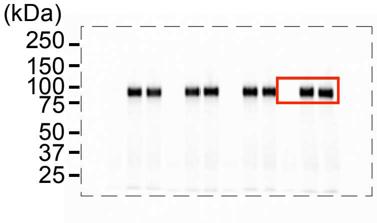

Figure 8e alpha-tubulin

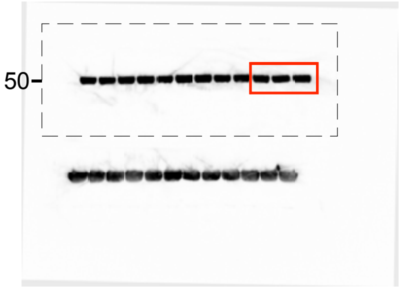

Supplementary Figure 10. Unprocessed blot images in Figs. 8c, e. Dotted squares indicate blotting membranes. Red squares indicate cropped sections.
