# Supplementary material for: αvβ3 Integrin induces partial EMT independent of TGF-β signaling
Source: Commun Biol. 2021 Apr 21;4:490. doi: 10.1038/s42003-021-02003-6 (PMC8060333; doi:10.1038/s42003-021-02003-6)
Supplement: Supplementary file 2 — Description of Additional Supplementary Files [file 42003_2021_2003_MOESM2_ESM.pdf]

Description of Additional Supplementary Files

File name: Supplementary Data 1

Description: The source data behind the graphs in the paper.
