# Supplementary material for: αvβ3 Integrin induces partial EMT independent of TGF-β signaling
Source: Commun Biol. 2021 Apr 21;4:490. doi: 10.1038/s42003-021-02003-6 (PMC8060333; doi:10.1038/s42003-021-02003-6)
Supplement: Supplementary file 4 — Reporting Summary [file 42003_2021_2003_MOESM4_ESM.pdf]

nature research

Corresponding author(s): Yoshinobu Kariya

Last updated by author(s): Mar 9, 2021

Reporting Summary

Nature Research wishes to improve the reproducibility of the work that we publish. This form provides structure for consistency and transparency in reporting. For further information on Nature Research policies, see our Editorial Policies and the Editorial Policy Checklist.

Statistics

For all statistical analyses, confirm that the following items are present in the figure legend, table legend, main text, or Methods section.

n/a	Confirmed	
[ ]	[x]	The exact sample size (*n*) for each experimental group/condition, given as a discrete number and unit of measurement
[ ]	[x]	A statement on whether measurements were taken from distinct samples or whether the same sample was measured repeatedly
[ ]	[x]	The statistical test(s) used AND whether they are one- or two-sided<br>*Only common tests should be described solely by name; describe more complex techniques in the Methods section.*
[x]	[ ]	A description of all covariates tested
[ ]	[x]	A description of any assumptions or corrections, such as tests of normality and adjustment for multiple comparisons
[ ]	[x]	A full description of the statistical parameters including central tendency (e.g. means) or other basic estimates (e.g. regression coefficient) AND variation (e.g. standard deviation) or associated estimates of uncertainty (e.g. confidence intervals)
[ ]	[x]	For null hypothesis testing, the test statistic (e.g. *F*, *t*, *r*) with confidence intervals, effect sizes, degrees of freedom and *P* value noted<br>*Give P values as exact values whenever suitable.*
[x]	[ ]	For Bayesian analysis, information on the choice of priors and Markov chain Monte Carlo settings
[x]	[ ]	For hierarchical and complex designs, identification of the appropriate level for tests and full reporting of outcomes
[ ]	[x]	Estimates of effect sizes (e.g. Cohen's *d*, Pearson's *r*), indicating how they were calculated

Our web collection on statistics for biologists contains articles on many of the points above.

Software and code

Policy information about availability of computer code

Data collection	Immunohistochemistry images were obtained using a Nanozoomer-SQ and NDPview2 software. qPCR data were acquired using StepOne and StepOne software. Western blotting images were acquired using Imager and software (#AE-9300H-CP, ATTO). Fluorescence images were obtained using an A1 confocal microscope and software (Nikon) or an IX71 fluorescence microscope and software (Olympus). FACS data were acquired using FACSCalibur and CellQuest software (BD Biosciences). Microscopy images were photographed using an IX71 phase contrast microscope (Olympus). Cell adhesion assay data were obtained using a microplate reader (BioRad, Model 680).
Data analysis	Most of data analysis and statistics were performed using GraphPad Prism Version 5.0a and SPSS statistics 26 softwares. FACS data analyses were performed with CellQuest software.

For manuscripts utilizing custom algorithms or software that are central to the research but not yet described in published literature, software must be made available to editors and reviewers. We strongly encourage code deposition in a community repository (e.g. GitHub). See the Nature Research guidelines for submitting code & software for further information.

Data

Policy information about availability of data

All manuscripts must include a data availability statement. This statement should provide the following information, where applicable:

- Accession codes, unique identifiers, or web links for publicly available datasets
- A list of figures that have associated raw data
- A description of any restrictions on data availability

The authors declare that all data supporting the findings in this study are included in the article and its supplementary Information files. Any additional information may be available from the corresponding author upon request. The co-expression between β3 integrin and αv integrin, vimentin, fibronectin, OVOL1, ZEB1, ZEB2, E-cadherin, ZO-1 in patients with lung adenocarcinoma was analyzed using RNA-Seq data (TCGA, https://cancergenome.nih.gov, Firehose Legacy, n = 517) and shown

in graphs using cBioPortal (https://www.cbioportal.org). The Kaplan-Meier analysis of overall survival was generated using the online source http://kmplot.com/analysis.

Field-specific reporting

Please select the one below that is the best fit for your research. If you are not sure, read the appropriate sections before making your selection.

☒ Life sciences ☐ Behavioural & social sciences ☐ Ecological, evolutionary & environmental sciences

For a reference copy of the document with all sections, see nature.com/documents/nr-reporting-summary-flat.pdf

Life sciences study design

All studies must disclose on these points even when the disclosure is negative.

Sample size	No statistical test was used to determine sample size.
Data exclusions	No data were excluded from analysis.
Replication	At least three independent experiments for all studies except for the experiments shown in Figs. 1b, 3g–k, 4b, which were performed once.
Randomization	All the animals were allocated randomly to the experimental and control groups.
Blinding	No blinded experiments except histological analyses were conducted in this study.

Reporting for specific materials, systems and methods

We require information from authors about some types of materials, experimental systems and methods used in many studies. Here, indicate whether each material, system or method listed is relevant to your study. If you are not sure if a list item applies to your research, read the appropriate section before selecting a response.

Materials & experimental systems

n/a	Involved in the study	
☐	☒	Antibodies
☐	☒	Eukaryotic cell lines
☒	☐	Palaeontology and archaeology
☐	☒	Animals and other organisms
☒	☐	Human research participants
☒	☐	Clinical data
☒	☐	Dual use research of concern

Methods

n/a	Involved in the study	
☒	☐	ChIP-seq
☐	☒	Flow cytometry
☒	☐	MRI-based neuroimaging

Antibodies

Antibodies used	mouse anti-E-cadherin (clone 36, #610182, BD Transduction Laboratories, 1:1000 WB), mouse anti-N-cadherin (clone 32, #610920, BD Transduction Laboratories, 1:1000 WB), mouse anti-αv integrin (clone 21, #611012, BD Transduction Laboratories, 1:1000 WB), mouse anti-β3 integrin (clone 1, #611140, BD Transduction Laboratories, 1:1000 WB), mouse anti-vimentin (clone V9, #sc-6020, Santa Cruz Biotechnology, 1:1000 WB), mouse anti-α-tubulin (clone DM 1A, #T9026, SIGMA, 1:5000 WB), mouse anti-FAK (clone 77, #610087, BD Transduction Laboratories, 1:1000 WB), mouse anti-phospho-FAK (clone 18, #611806, BD Transduction Laboratories, 1:1000 WB), rabbit anti-ZEB1 (clone D80D3, #3396, Cell Signaling Technology, 1:1000 WB), rabbit anti-Smad2/3 (clone D7G7, #8685, Cell Signaling Technology, 1:1000 WB, 1:100 IF), rabbit anti-phospho-Smad2 (clone 138D4, #3108, Cell Signaling Technology, 1:1000 WB), rabbit anti-phospho-Smad3 (clone C25A9, #9520, Cell Signaling Technology, 1:1000 WB). rabbit anti-ZO-1 (#GTX108592, Gene Tex, 1:1000 WB), rabbit anti-OVOL-1 (#14082-1-AP, Proteintech, 1:1000 WB), mouse anti-αvβ3 integrin (clone LM609, #MAB1976, Merck Millipore, 1:100 IF), mouse anti-E-cadherin (clone HECD1, #M106, Takara, 1:2000 IF), goat anti-vimentin (clone C-20, #sc-7557, Santa Cruz Biotechnology, 1:100 IF), rat anti-CD31 (clone MEC13.3, #553370, BD Transduction Laboratories, 1:100 IF), mouse anti-αvβ3 integrin (clone 23C6, #304402, BioLegend 1:100 FACS, 1:100 Function blocking), rabbit anti-β3 integrin (#ab179473, abcam, 1:800 IHC), mouse anti-Ki67 (#610968, BD Transduction Laboratories, 1:100 IF).
Validation	Validation data for each antibody used in this study are available on the manufacturer's website.

Eukaryotic cell lines

Policy information about cell lines

Cell line source(s)	Human lung cancer cell lines A549 and human embryonic kidney cell lines 293T were obtained from the RIKEN BRC through the National Bio-Resource Project of the MEXT, Japan. Modified human 293 phoenix cells were a gift from Dr. M Peter

	Marinkovich (Stanford University, Stanford, CA). Human lung cancer cell lines, H358, H460 and H1975 cells were purchased from the American Type Culture Collection (ATCC).
Authentication	A549, 293T were directly obtained from the RIKEN BRC. H358, H460 and H1975 cells were directly obtained from ATCC. 293 phoenix cells were not authenticated.
Mycoplasma contamination	All cell lines were tested negative for mycoplasma contamination.
Commonly misidentified lines (See ICLAC register)	No cell lines in this study are listed in the database of commonly misidentified lines.

Animals and other organisms

Policy information about studies involving animals; ARRIVE guidelines recommended for reporting animal research

Laboratory animals	6- to 7-weeks-old female nude mice (BALB/c nu/nu, SLC).
Wild animals	This study does not involve wild animals.
Field-collected samples	This study does not involve samples collected from fields.
Ethics oversight	All animal studies were performed in accordance with protocols approved by Fukushima Medical University Animal Care and Use Committee.

Note that full information on the approval of the study protocol must also be provided in the manuscript.

Flow Cytometry

Plots

Confirm that:

- [x] The axis labels state the marker and fluorochrome used (e.g. CD4-FITC).
- [x] The axis scales are clearly visible. Include numbers along axes only for bottom left plot of group (a 'group' is an analysis of identical markers).
- [x] All plots are contour plots with outliers or pseudocolor plots.
- [ ] A numerical value for number of cells or percentage (with statistics) is provided.

Methodology

Sample preparation	For analyzing cell surface expression of αvβ3 integrin, the cells were detached from a dish using 0.25% trypsin/PBS with 1 mM EDTA. After quenching trypsinization with a medium that contained 10% FBS, the cells were washed twice with PBS that contained 1 mM EDTA (PBS/EDTA), and then suspended in PBS/EDTA. The cells were incubated with a mouse monoclonal antibody against αvβ3 integrin (clone 23C6, #304402, BioLegend 1:100) on ice for 30 min. After washing once with PBS/EDTA, the cells were incubated with an Alexa Fluor 488-conjugated goat secondary anti-mouse IgG antibody (1:500). After incubation on ice for 15 min, the cells were washed three times with PBS/EDTA, and then analyzed by flow cytometry using FACSCalibur and CellQuest software (BD Biosciences).
Instrument	FACSCalibur (BD Biosciences)
Software	CellQuest software (BD Biosciences)
Cell population abundance	At least 10,000 events were analyzed for each sample.
Gating strategy	The data in this study were derived from all cells without gating.

- [ ] Tick this box to confirm that a figure exemplifying the gating strategy is provided in the Supplementary Information.
